# *MALAT1* promotes malignancy of HBV-related hepatocellular carcinoma by regulating IGF2BP3-mediated nuclear-cytoplasmic shuttling

**DOI:** 10.7150/ijbs.112133

**Published:** 2025-07-28

**Authors:** Ze-Bang Du, Xin-Mou Wu, Tun Han, Yu-Xin Cai, Bo Qian, Yu-Shi Shen, Han-Yu Zhang, Jia-Shen Wu, Jie He, Xiao-Xuan Chen, Dong-Bei Guo, Hang-Tian Zhong, Xiong Li, Lei Zhang, Xiao-Ming Luo, Wen-Gang Li, Yu-Chun Lin, Zhong-Ning Lin

**Affiliations:** 1 State Key Laboratory of Vaccines for Infectious Diseases, Xiang An Biomedicine Laboratory, Xiang'an Hospital of Xiamen University, National Innovation Platform for Industry-Education Integration in Vaccine Research, School of Public Health, Xiamen University, Xiamen 361102, China; 2 Department of Preventive Medicine, School of Public Health, Chengdu Medical College, Chengdu 610500, China.; 3 Department of Hepatobiliary Surgery, Cancer Research Center, Xiang'an Hospital of Xiamen University, School of Medicine, Xiamen University, Xiamen, Fujian 361102, China.

**Keywords:** HBV-related HCC, *MALAT1*, m6A modification, nuclear-cytoplasmic shuttling, IGF2BP3, anti-HBx transposon plasmids

## Abstract

Hepatitis B virus (HBV) X protein (HBx) plays a critical role in the progression of HBV-related hepatocellular carcinoma (HCC). Long non-coding RNAs (lncRNAs) regulate various biological processes and contribute to HCC development, with their therapeutic potential in disease progression recently gaining significant attention. However, the involvement of lncRNAs in HBx-related hepatocarcinogenesis and the underlying regulatory mechanisms remain unclear. In this study, we conducted a comprehensive analysis of multi-database sequencing data to identify metastasis-associated lung adenocarcinoma transcript 1 (*MALAT1*) as an HBx-associated lncRNA and observed its upregulation in HBV-related HCC tissues and cells upon HBx expression. Additionally, high *MALAT1* expression was correlated with poor prognosis and advanced HCC progression. *MALAT1* overexpression significantly promoted the proliferation, migration, and invasion of HCC cells. Mechanistic investigations revealed that *MALAT1* was transported to the cytoplasm and enhanced RNA stability in a N6-methyladenosine (m6A)-dependent manner through direct interaction with and recruitment of insulin-like growth factor 2 mRNA-binding protein 3 (IGF2BP3). Targeting *MALAT1 in vivo* with antisense oligonucleotides (ASO)-*MALAT1* treatment effectively suppressed the progression of xenograft tumors and orthotopic liver tumors in HBx-related HCC. Moreover, hydrodynamic-based gene delivery (HGD) was utilized to introduce anti-HBx transposon plasmids into murine hepatocytes, thereby suppressing *MALAT1*-m6A-mediated HBV-related hepatocarcinogenesis in HBx transgenic (HBx-Tg) mice. Overall, our findings shed novel light on the regulatory role of IGF2BP3-mediated *MALAT1* nuclear-cytoplasmic shuttling and RNA stabilization via m6A modification during HCC progression. These results suggest that m6A-based* MALAT1* expression serves as a novel diagnostic and prognostic biomarker for targeted epigenetic intervention in HBV-related HCC.

## Introduction

Hepatocellular carcinoma (HCC) is one of the most prevalent and lethal malignancies worldwide, posing a significant challenge to public health with a growing incidence rate 1. The primary risk factor for HCC development, identified in 80%-90% of cases, is chronic hepatitis B virus (HBV) infection, which often progresses to end-stage liver diseases such as cirrhosis and ultimately leads to HCC 2. Chronic HBV infection contributes to hepatocarcinogenesis through both direct mechanisms, including gene integration, mutation-induced genomic instability, and activation of cancer-related signaling pathways, and indirect mechanisms involving viral proteins 3. Among the viral proteins encoded by the HBV genome, the HBV X protein (HBx) is a multifunctional regulatory protein that plays a critical role in initiating and progressing HCC by activating various oncogenes 4, 5. However, the epigenetic mechanisms by which HBx contributes to HCC pathogenesis remain poorly understood.

Accumulating evidence has demonstrated that HBx plays a critical role in the pathogenesis of HCC by inducing genetic alterations in tumor suppressor genes and oncogenes. Additionally, epigenetic aberrations are also pivotal in the pathogenesis of HCC 4, 6. Recent studies have highlighted the dysregulation of long non-coding RNAs (lncRNAs) in HBV-related HCC and HBV/HBx-expressing cells. These lncRNAs, which are longer than 200 nucleotides and lack protein-coding capacity 7, are involved in a wide range of cellular processes, including chromatin modification, transcriptional and post-transcriptional regulation, and function as signals, decoys, guides, scaffolds, and competing endogenous RNAs (ceRNAs) 8, 9. Dysregulated lncRNAs, such as *HOTAIR*, have been associated with diseases like liver fibrosis 10, liver cancer 11, and metastasis 12, and serve as early prognostic indicators for lower survival rates. These findings suggest that lncRNAs can act as molecular markers and prognostic indicators for HCC due to their abnormal expression and specific molecular functions. Importantly, emerging evidence indicates that lncRNAs mediate interactions between host cells and viruses, including HBV. For example, *HULC* enhances the stability of HBV covalently closed circular DNA (cccDNA) 13, while the HBx-bound lncRNA *DLEU2* sustains the transcription of cccDNA and cancer-relatd genes 14. Moreover, HBx-upregulated *TRERNA1* has been shown to confer resistance to sorafenib in HCC cells 15. Collectively, these studies imply that differential lncRNA expression is regulated by HBV infection and HBx expression, and that HBx significantly contributes to the lncRNA-associated epigenetic regulation of hepatocytes in HBV-related HCC. Therefore, identifying functional lncRNAs targeted by HBx is crucial for developing novel therapeutic strategies against HBV-related HCC.

N6-methyladenosine (m6A) modification, constituting 0.1%-0.4% of native cellular RNA adenosine residues, is a prevalent epigenetic mark across various RNA types 16. This internal modification extensively regulates gene expression by modulating RNA processing, localization, translation, and decay. These processes are orchestrated by three categories of proteins: "writers", "erasers", and "readers". m6A modification is also widely present in lncRNAs and regulates their functions, including nuclear export, potential peptide translation, and stability. However, the role of m6A in regulating HBx-induced lncRNAs in HBV-related HCC remains elusive.

In this study, we identified metastasis-associated lung adenocarcinoma transcript 1 (*MALAT1*) as an lncRNA that is upregulated in HBV/HBx-expressing cells, HBx transgenic (HBx-Tg) mice, and patients with HBV-related HCC. We further investigated the biological function of *MALAT1* and its m6A regulation in hepatocarcinogenesis both *in vitro* and *in vivo*. Mechanistically, we explored the m6A methylation status of *MALAT1* at specific sites (A6021 and A7265) and the role of insulin-like growth factor 2 mRNA-binding protein 3 (IGF2BP3) in mediating *MALAT1* nuclear-cytoplasmic shuttling and RNA stabilization. Our findings provide new insights into the mechanisms of m6A-based lncRNAs in HBV-related HCC and highlight the potential of m6A-modified lncRNAs as novel epigenetic biomarkers and theranostic targets.

## Materials and Methods

### HCC tissue specimens

Twelve human HCC tumor tissues and corresponding peritumor tissues were obtained from patients who underwent liver tumor resection at Xiang'an Hospital of Xiamen University (Xiamen, China). Immediately upon retrieval, all samples were frozen in liquid nitrogen and stored at -80 °C until use. The levels of *MALAT1* were detected via RNA fluorescence *in situ* hybridization (FISH) assays. Some samples were fixed and paraffin-embedded for histological examination. Protein levels, including HBx, IGF2BP3, E-cadherin, and Vimentin, were detected by western blotting (WB) and immunohistochemistry (IHC) staining. Written informed consent was obtained from all HCC patients involved in this study, and the study was approved by the Ethics Committee of Xiamen University.

### *In vivo* xenograft tumor study

An *in vivo* xenograft tumor study was conducted following a previously described protocol 17, 18. To explore the effects of HBx expression and *MALAT1* genetic targeting intervention on the progression of HCC cell xenograft tumors, BALB/c nude mice (4- to 6-week-old) were randomly divided into four groups (n = 8 per group) as follows: Ctrl (HepG2-pc3.1-NC), HBx (HepG2-pc3.1-HBx), HBx+ASO-NC, and HBx+ASO-*MALAT1*. After allowing the xenograft tumors to grow for one week, the HBx+ASO-NC and HBx+ASO-*MALAT1* groups were injected with ASO-NC or ASO-*MALAT1* (5 nmol per injection, once every 2 days, for a total of 7 injections) via the tail vein. The mice were sacrificed on the day of the last injection. Tumor width (W), tumor length (L), and body weight were measured. Tumor volume (V) was calculated using the formula: V= (W^2^×L)/2. Xenograft tumors were collected for RNA FISH, quantitative real-time PCR (qRT-PCR), WB, hematoxylin and eosin (HE) staining, and IHC assays. All animal experiments were approved by the Experimental Animal Ethics Committee of Xiamen University (Ethics Approval No. XMULAC20220282, dated 2022-03-12).

### Orthotopic liver tumor model

To establish an orthotopic liver tumor model mimicking HBV-related HCC *in vivo*, luciferase-expressing HepG2.2.15 cells (HepG2.2.15-Luc) were used. Briefly, 14 male nude mice (7-8 weeks old, average weight 20 g) were anesthetized with 1.5-2% isoflurane. After cleaning the surgical area with iodine and alcohol, a 1-1.5 cm midline incision was made below the xiphoid process. A total of 50 µl of a suspension containing 5×10^5^ HepG2.2.15-Luc cells mixed with Matrigel was slowly injected into the top lobe of the liver, which was gently exteriorized through the incision. The liver was then carefully replaced, and the wound was sutured. The mice were monitored until they fully recovered from anesthesia and were then returned to their cages. Humane endpoints were defined as 20% body weight loss, loss of mobility, or significant decrease in activity. However, no mice reached these endpoints during the study.

The tumor-bearing mice were randomly divided into two groups (n = 7 per group): ASO-NC and ASO-*MALAT1*. The ASO-*MALAT1* group received intravenous injections of 10 nmol ASO-*MALAT1* on days 1, 3, and 5, while the ASO-NC group received equivalent doses of ASO-NC as a negative control (NC). Tumor progression was monitored noninvasively using bioluminescence imaging (BLI) on days 7, 14, 21, and 28 post-implantation. Imaging was performed using an IVIS Spectrum system (PerkinElmer, Inc., Waltham, MA, USA) following intraperitoneal injection of D-luciferin (150 mg/kg). After 4 weeks, the animals were sacrificed, and their livers were harvested for macroscopic tumor assessment, histological examination, and molecular analysis. Intrahepatic tumor burden and abdominal dissemination were recorded.

### HBx transgenic (HBx-Tg) mouse study

HBx-Tg mice were generated as previously described 19, 20. For the RNA sequencing analysis, livers were collected from HBx-Tg mice (n = 4) and wild-type (WT) mice (n = 4). Eight cDNA libraries were constructed using 3 μg of RNA per liver sample. The quality of the libraries was assessed using the Agilent Bioanalyzer 2100 system. The libraries were sequenced at the Novogene Bioinformatics Institute (Beijing, China) on an Illumina HiSeq 4000 platform, generating 150 bp paired-end reads after clustering of the index-coded samples. Data were extracted from the resulting images using Agilent's Feature Extraction Software (Agilent Technologies; Santa Clara, CA, USA).

To suppress HBx expression in the livers of HBx-Tg mice, *in vivo* gene delivery was performed via intravenous injection of PB-20F3 transposon plasmids expressing the anti-HBx antibody (provided by Prof. Quan Yuan, Xiamen University) using TransIT-EE Hydrodynamic Delivery Solution (TaKaRa; Kyoto, Japan). Injections were administered through the tail vein at a constant speed and completed within 10 seconds. For long-term gene expression experiments, HBx-Tg mice were injected with 0.3 mL of TransIT-EE containing PB-20F3 (10 μg) and PB-Trans (5 μg) (a universal plasmid containing PBase transposase) once every 4 days for a total of 7 injections. After the final injection, both the WT and HBx-Tg mice were sacrificed, and their livers were collected for tissue lysate extraction and serial sectioning. All animal procedures were ethically approved by the Experimental Animal Ethics Committee of Xiamen University (Ethics Approval No. XMULAC20220282, dated 2022-03-12).

Other materials and methods are described in the Supplementary files.

## Results

### HBx expression-upregulated* MALAT1* is positively correlated with poor prognosis in HCC patients

**Figure 1:**
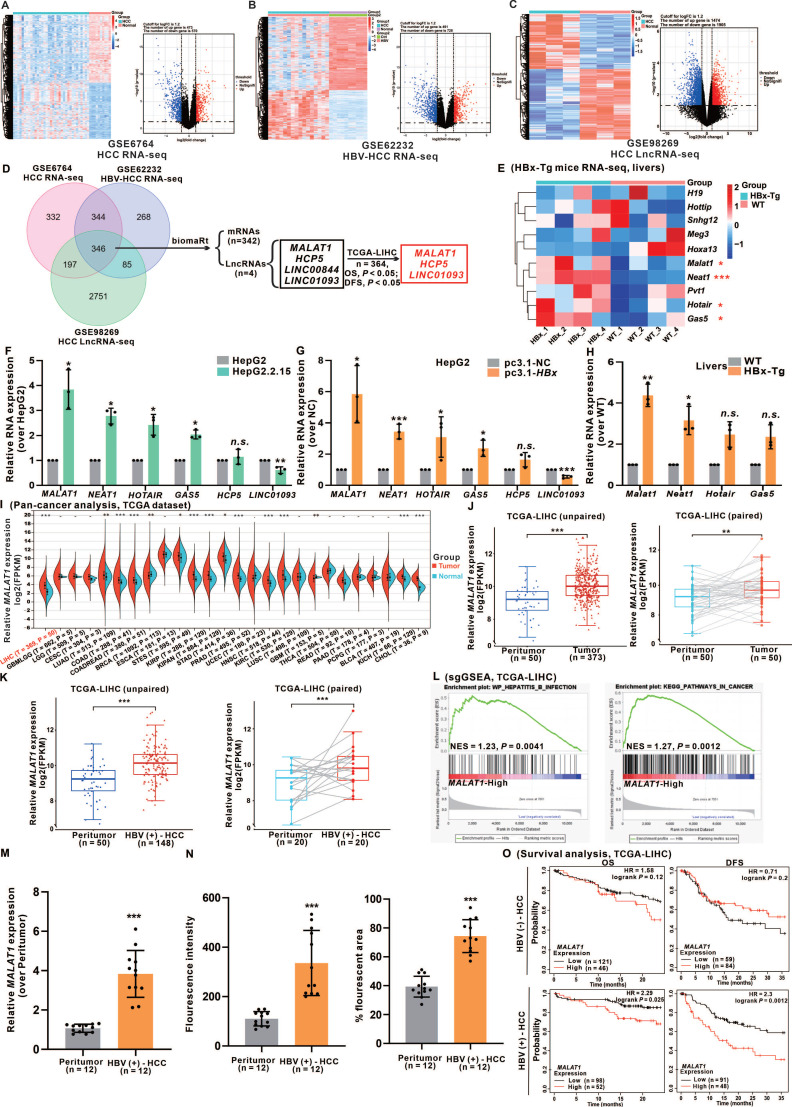
** HBx expression-upregulated *MALAT1* is positively correlated with poor prognosis in HCC patients. A-C** Three HCC gene expression datasets, including GSE6764 (n = 45), GSE62232 (n = 26), and GSE98269 (n = 6), were retrieved from the GEO database. The raw data were normalized, and the numbers of DEGs (*P* < 0.05 and |log2FC| ≥ 1.2) were 1043, 1219 and 3379, respectively, as shown in the heatmap and volcano plots. **D** Venn diagram showing the overlap between the DEGs of the three cohorts. **E** Heatmap of 10 HBx-related HCC-associated lncRNAs in HBx-Tg mice (n = 4) and WT mice (n = 4). **F-G** Relative expression of 7 candidate lncRNAs was detected via qRT-PCR in HBV/HBx-expressing HCC cells. **H** Relative expression of 7 candidate lncRNAs was detected via qRT-PCR in HBx-Tg mice livers. **I** Pan-cancer analysis of *MALAT1* expression in the TCGA-LIHC cohort. **J-K** Quantification of *MALAT1* expression in HBV-infected and uninfected HCC tissue and peritumor tissue samples. **L** GSEA of *MALAT1* in the TCGA-LIHC cohort. NES, normalized enrichment score. **M-N** Paired tumor and adjacent peritumor liver tissues were collected from 12 patients with HBV-related HCC.** M** Relative expression of *MALAT1* was detected by qRT-PCR.** N** Quantification of FISH results showing fluorescence intensity and percentage of *MALAT1*-positive area. **O** Kaplan-Meier analysis of the OS and DFS of patients with *MALAT1*, with or without hepatitis infection in the TCGA-LIHC cohort. HR, hazard ratio. *P < 0.05; **P < 0.01; ***P < 0.001. *n.s.*, not significant.

To identify differentially expressed lncRNAs associated with HBV-related HCC, we extracted three HCC gene expression datasets from the GEO database. Employing the criteria of *P* < 0.05 and |log2FC| ≥ 1.2, we identified 1043, 1219, and 3379 differentially expressed genes (DEGs) from GSE6764, GSE62232, and GSE98269, respectively, which included both HCC tumor tissues and healthy liver controls. Heatmaps and volcano plots were generated to visualize the DEGs of mRNAs and lncRNAs in patients with HCC (Fig. 1A-C). By intersecting the three cohorts, we identified 346 significantly altered DEGs. Subsequently, we screened 4 lncRNAs for survival analysis using the overall survival (OS) and disease-free survival (DFS) data from the TCGA-LIHC cohort. After survival analysis, we successfully identified 3 candidate lncRNAs (Fig. 1D).

Furthermore, considering the deregulation of multiple lncRNAs in HBV infection and HBV-related HCC patients 21, 22, we evaluated 9 known HBV-related HCC-modulated lncRNAs in the livers of HBx-Tg and WT mice livers via RNA-seq analysis (Fig. 1E). Based on the above results, we successfully identified 6 HBx-associated candidate lncRNAs (*MALAT1*, *NETA1*, *HOTAIR*, *GAS5*,* HCP5*, and* LINC01093*). These lncRNAs were significantly differentially expressed between tumor and normal tissues and were significantly correlated with both the OS and DFS of HCC patients (Fig. S1A). Validation of these candidate lncRNAs' expression levels was performed in HBV/HBx-expressing HCC cells and the livers of HBx-Tg mice (Fig. 1F-H). Based on the expression data, *MALAT1*, which exhibited the highest abundance, displayed a dose- and time-dependent relationship with HBx expression, suggesting that further investigation is warranted (Fig. S1B-C). To further determine whether the induction of *MALAT1* is uniquely driven by HBx rather than by other HBV components, we transfected HepG2 cells with a full-length HBV plasmid, an HBx-deficient (HBV-x-null) plasmid, and an HBx-expressing plasmid alone. Only the presence of HBx significantly upregulated *MALAT1* expression, while the x-null construct had no effect (Fig. S1D). Additionally, individual overexpression of HBV-encoded proteins (HBs, HBc, HBp, and HBx) revealed that only HBx was capable of inducing *MALAT1* upregulation, further confirming HBx as the sole viral component responsible for this effect (Fig. S1E-F). GO and KEGG enrichment analyses of RNA-Seq data from HBx-Tg mice livers revealed the activation of oncogenic pathways (Fig. S1G-H), which is consistent with the role of HBx in the carcinogenesis of HCC.

To elucidate the oncogenic relevance of *MALAT1*, a pan-cancer analysis was conducted, revealing that *MALAT1* is significantly overexpressed across various tumor types compared with corresponding peritumor tissues (Fig. 1I). Examination of TCGA-LIHC RNA-seq data revealed *MALAT1* upregulation in both unpaired and paired HCC tissues, particularly in association with HBV infection (Fig. 1J-K). Single-gene gene-set enrichment analysis (sgGSEA) further highlighted the strong association between *MALAT1* and HBV infection, as well as with cancer-related signaling pathways (Fig. 1L). To further evaluate the specificity of *MALAT1* in HBV-related HCC, we extended our analysis to publicly available GEO datasets covering multiple HCC etiologies. Specifically, *MALAT1* expression was examined in 7 HBV-HCC datasets, 7 HCV-HCC datasets, 4 NAFLD-related HCC datasets, and 2 datasets from patients with alcoholic liver disease (ALD) (Fig. S2A-B). *MALAT1* was consistently upregulated in 5 out of 7 HBV-HCC datasets (log2FC > 1), while no significant or consistent change was observed in HCV- or NAFLD-related HCC. Moreover, ALD patient samples showed no significant difference in *MALAT1* expression compared with healthy controls (Fig. S2A-B), supporting the notion that *MALAT1* upregulation is preferential to HBV-driven hepatocarcinogenesis. In addition to public datasets, we validated *MALAT1* expression in an independent clinical cohort of 12 patients with HBV-related HCC from our collaborating hospital. qRT-PCR and FISH performed on paired tumor and adjacent liver tissues confirmed significantly elevated *MALAT1* expression in tumor tissues (Fig. 1M-N, S2C), further reinforcing the robustness and translational relevance of our findings. Consistent patterns of upregulation were also observed in the livers of HBx-Tg mice (Fig. S2D), supporting the functional relevance of HBx in *MALAT1* activation. Furthermore, survival analysis of the TCGA-LIHC cohort stratified by HBV status indicated that *MALAT1* overexpression was significantly associated with reduced OS and DFS exclusively in HBV-positive patients (Fig. 1O). These prognostic implications were further corroborated by the predictive accuracy of *MALAT1* in a nomogram-based model (Fig. S2E). Collectively, these results underscore the specificity and prognostic value of *MALAT1* in HBV/HBx-related HCC and support its potential utility as a biomarker for early detection and risk stratification.

### *MALAT1* promotes the proliferation and metastasis of HBV/HBx-expressing HCC cells *in vitro*

The elevated *MALAT1* expression in HCC patients prompted an investigation into its functional role in HBV-related HCC progression. Among the seven HBx-expressing HCC cell lines, HepG2 cells presented the most substantial increase in HBx-upregulated *MALAT1* expression (Fig. S3A). Consequently, HepG2 and HepG2.2.15 cells, which harbor the HBV genome, were selected for further investigation. To achieve stable *MALAT1* overexpression, two strategies were employed. The initial approach involved transient transfection of the pCDH-*MALAT1* plasmid, resulting in a modest threefold increase (Fig. S3B). However, due to the large size of the full-length *MALAT1* transcript (8779 bp), further enhancement of expression was limited. Subsequently, CRISPR/Cas9 technology was utilized to construct dCas9-*MALAT1* plasmids for transfection into HBV/HBx-expressing HCC cells, ensuring stable and upregulated *MALAT1* expression through the dCas9-SAM system (Fig. S3C) 23, 24. This strategy, which is more effective for achieving high lncRNA expression levels and stable cell lines, circumvents limitations related to transcript length. For *MALAT1* silence, ASOs and CRISPR/Cas9 technology were employed, both of which demonstrated efficient silence (Fig. S3D-E). Considering the potential off-target effects associated with Cas9-*MALAT1*, ASO-*MALAT1* has emerged as the preferred approach for subsequent intervention, holding promise in contemporary drug development.

**Figure 2:**
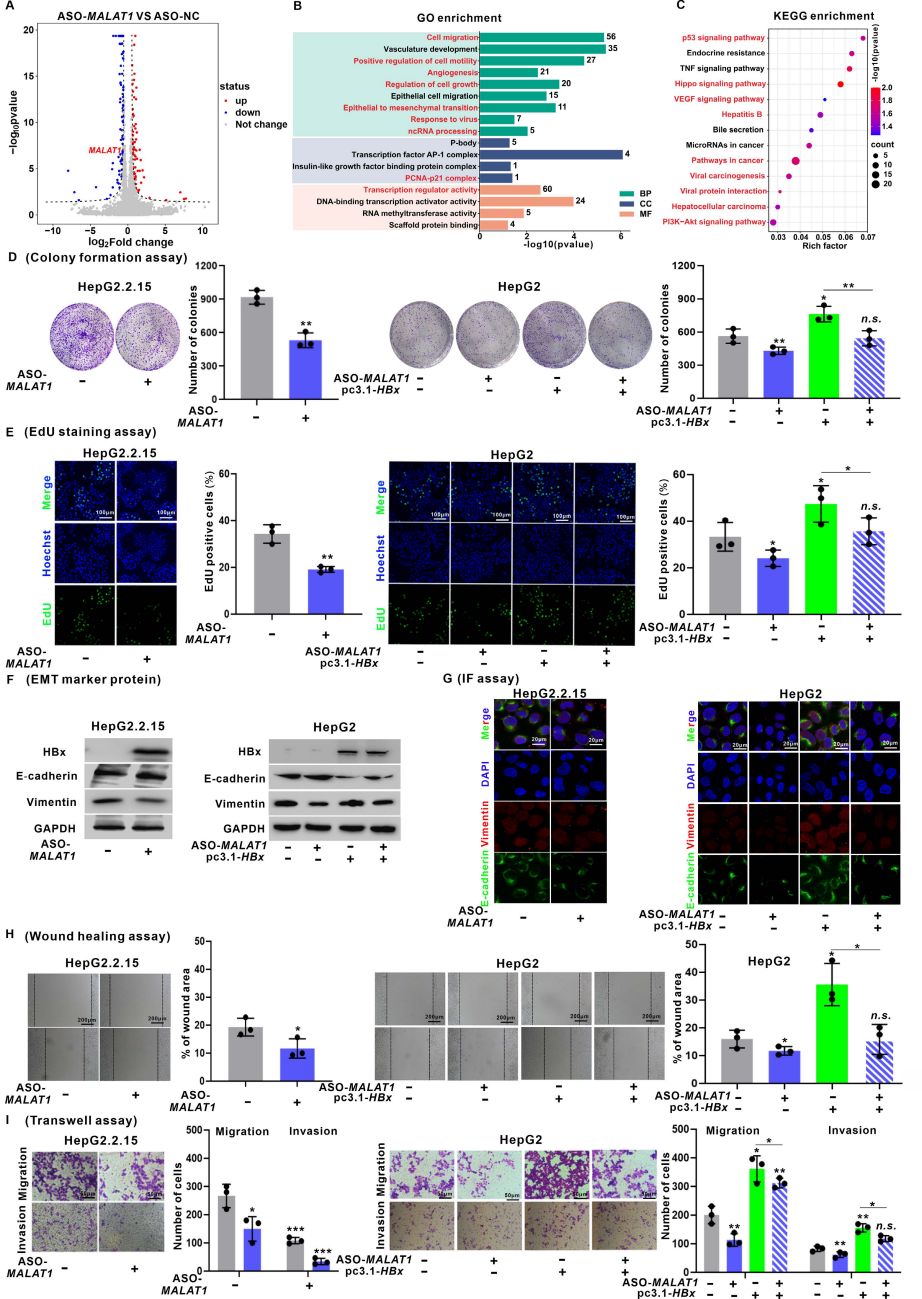
***MALAT1***** promotes the proliferation and metastasis of HBV/HBx-expressing HCC cells *in vitro*. A-C** HepG2.2.15 cells were transfected with ASO-*MALAT1* (100 nM, for 24 h) to construct *MALAT1-*silence cells, while ASO-NC was used as a negative control (NC). RNA sequencing was performed for transcriptomic profiling analysis in cells.** A** Volcano plot illustrating DEGs. **B** GO enrichment analysis of DEGs. **C** KEGG enrichment analysis of DEGs. **D-I** HepG2.2.15 and HBx-expressing HepG2 cells were transfected with ASO-*MALAT1* (100 nM, for 24 h), while ASO-NC was used as a control. **D** Colony formation assays were performed to determine the clonogenicity of the cells. The relative number of colonies is shown.** E** Cell proliferation was detected using EdU staining (green). Nuclei were counterstained with Hoechst 33342 (blue). Scale bar: 100 μm. **F-G** The levels of E-cadherin and Vimentin were detected by WB (F) and IF staining (G). Scale bar: 20 μm. **H** Cell migration was measured by wound healing assays.** I** The migration and invasion of the cells were examined via Transwell assays. *P < 0.05; **P < 0.01; ***P < 0.001. *n.s.*, not significant.

To elucidate the downstream molecular mechanisms governed by *MALAT1* in HBV-related HCC, transcriptome profiling was performed in HepG2.2.15 cells following *MALAT1* silence. Differential gene expression analysis (|log2FC| > 1, *P* < 0.05) confirmed successful silencing of *MALAT1* (log2FC = -1.2, *P* < 0.05) and identified a set of significantly regulated genes (Fig. 2A). GO enrichment analysis revealed that these DEGs were predominantly associated with "Cell migration", "Angiogenesis", "Regulation of cell growth", "Epithelial to mesenchymal transition (EMT)", and "ncRNA processing" (Fig. 2B). KEGG analysis further indicated enrichment in the pathways such as "P53 signaling", "Hippo signaling", "VEGF signaling", and "Hepatitis B" (Fig. 2C). Moreover, GSEA demonstrated significant suppression of oncogenic processes such as "Viral hepatitis", "Cell growth regulation", "EMT", and "P53 signaling" in *MALAT1*-silenced cells (Fig. S3F-I). Collectively, these findings demonstrate that *MALAT1* functions as a key oncogenic lncRNA in HBV-related HCC by orchestrating a broad network of cancer-associated signaling pathways. This regulatory role is consistent with *MALAT1*'s known pro-metastatic functions and provides novel insights into its HBx-specific oncogenic mechanisms.

Consistent with these transcriptomic changes, cell viability assays revealed that *MALAT1* silence suppressed proliferation, whereas *MALAT1* overexpression promoted proliferation in HBV/HBx-expressing HCC cells (Fig. S3J-K). This observation was further corroborated by colony formation and EdU assays (Fig. 2D-E, Fig. S4A-B). Additionally, a marked decrease in the epithelial marker E-cadherin was detected upon *MALAT1* silence, while an increase was observed with *MALAT1* overexpression. Conversely, the expression of the mesenchymal marker Vimentin decreased with *MALAT1* silence but increased with *MALAT1* overexpression (Fig. 2F-G, Fig. S4C-D). Wound healing and Transwell assays demonstrated that *MALAT1* silence notably reduced the migratory and invasive capacities, whereas *MALAT1* overexpression enhanced these abilities in HBV/HBx-expressing HCC cells (Fig. 2H-I, Fig. S4E-F). These findings illustrate the oncogenic role of *MALAT1* in promoting the proliferation and metastasis abilities of HBV-associated and HBx-expressing HCC cells.

Interestingly, beyond its impact on tumor progression, *MALAT1* silencing also affected HBV replication. Quantification of virological parameters after ASO-*MALAT1* treatment showed that while HBsAg and HBeAg levels remained largely unchanged (Fig. S5A-B), pgRNA, intracellular HBV DNA, and relaxed circular cccDNA (rcccDNA) levels were significantly reduced (Fig. S5C-E). These findings suggest a previously unrecognized role for *MALAT1* in modulating HBV transcription and replication, possibly through regulation of viral RNA stability or chromatin-related transcriptional machinery. This expands the functional significance of *MALAT1* beyond tumorigenesis, revealing its dual involvement in both hepatocarcinogenesis and viral persistence.

### The m6A level of *MALAT1* is upregulated by the recruitment of the m6A methyltransferase complex

**Figure 3:**
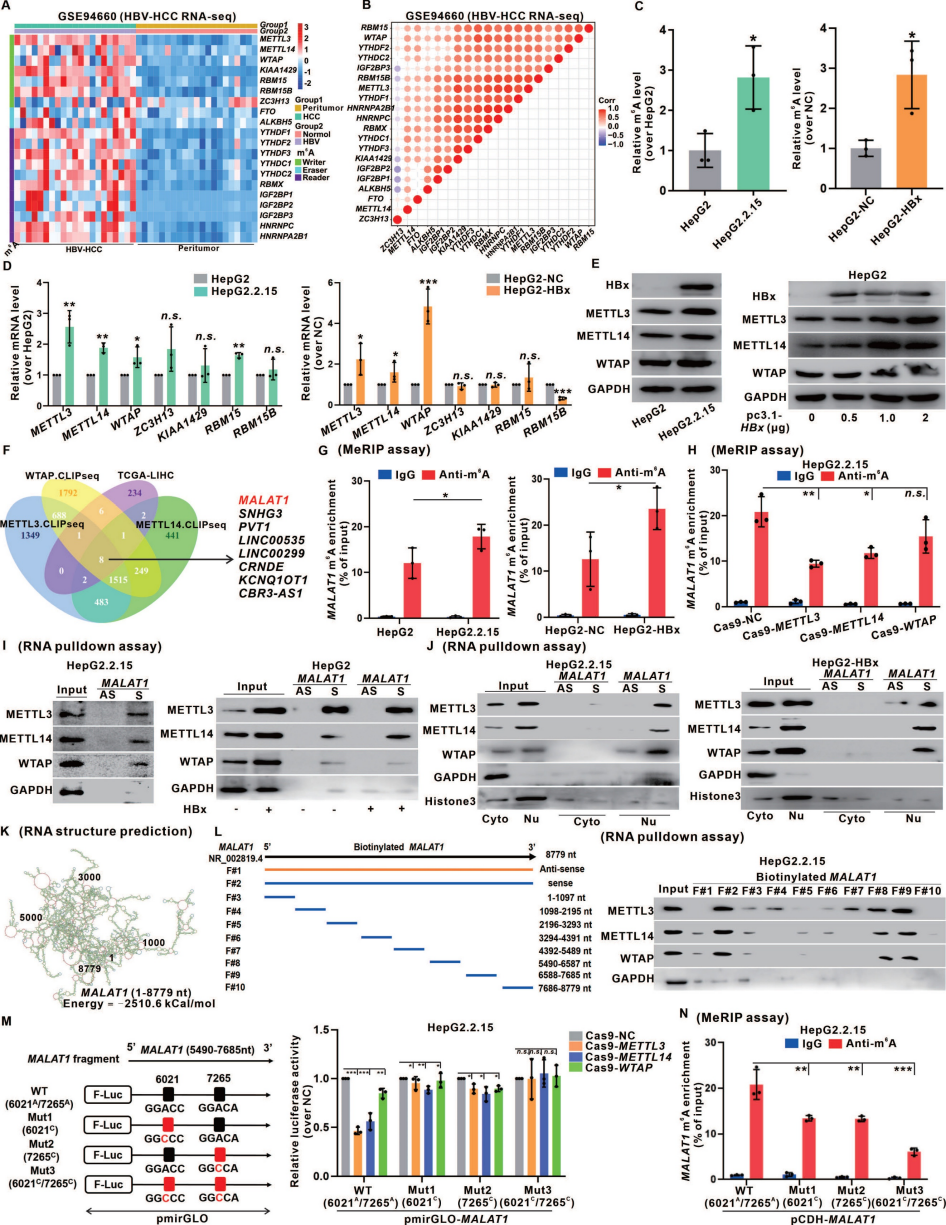
**The m6A level of *MALAT1* is upregulated by the recruitment of the m6A methyltransferase complex. A** Heatmap of 20 m6A-related genes in the livers of HBV-HCC patients (n = 21) and peritumor (n = 21) tissues. **B** Pearson's correlation analysis was used to determine the relationships between the mRNA levels of 20 m6A-related genes. **C** The overall m6A content in HepG2.2.15 and HepG2 cells was analyzed by m6A quantitation analysis. **D** Relative expression of the 7 m6A methyltransferase genes was detected by qRT-PCR in HepG2.2.15 and HepG2 cells. **E** The protein levels of METTL3, METTL14, and WTAP in HepG2.2.15 and HepG2 cells were detected by WB. **F** Venn diagram showing the overlap of METTL3-, METTL14-, and WTAP-bound transcripts revealed by CLIP-seq and the TCGA-LIHC cohort DEGs. **G** MeRIP of *MALAT1* transcripts in HepG2.2.15 and HepG2 cells upon indicated knockdown. The abundance of *MALAT1* among MeRIP with anti-m6A antibodies was measured via qPCR and normalized to that of IgG. **H** MeRIP of *MALAT1* transcripts in HepG2.2.15 cells. **I** METTL3, METTL14, and WTAP were pulled down by biotin-labeled sense *MALAT1* (S) but not *MALAT1* antisense (AS) RNA in HepG2.2.15 and HepG2 cells. **J** METTL3, METTL14, and WTAP were pulled down in cytoplasmic and nuclear fractions of HepG2.2.15 and HepG2 cells. **K** *MALAT1* secondary structure was predicted by lnCAR. **L** Scheme of full-length biotinylated-*MALAT1* (S#2), antisense *MALAT1* (S#1), and 8 truncated biotinylated-*MALAT1* fragments based on the sub-structures: S#3(1-1097 nt), S#4 (1098-2195 nt), S#5 (2196-3293 nt), S#6 (3294-4391 nt), S#7 (4392-5489 nt), S#8 (5490-6587 nt), S#9 (6588-7685 nt) and S#10 (7686-8779 nt). The full-length biotinylated-*MALAT1* and its truncations were separately transfected into HepG2.2.15 cells. **M** Dual-luciferase reporter assays were used to confirm the interaction between METTL3, METTL14, WTAP and 2 mutations (6021^C^ and 7265^C^) of the *MALAT1* m6A sites. **N** MeRIP of *MALAT1* transcripts in HepG2.2.15 cells transfected with *MALAT1* mutant plasmids. *P < 0.05; **P < 0.01; ***P < 0.001. *n.s.*, not significant.

Recent studies on tumor epigenetics have shed light on the impact of m6A modification on lncRNAs 25. To investigate the potential influence of m6A modification on *MALAT1* regulation in HCC, we analyzed transcriptomic data from HBV-related HCC patients and normal individuals from the GEO database (GSE94660) and identified 7 "writers", 2 "erasers", and 11 "readers" of m6A modification on the basis of prior research 26. A heatmap revealed significant m6A methylation activation in the livers of patients with HBV-related HCC (Fig. 3A), with a positive correlation between the relative expression levels of these genes (Fig. 3B). Comparable results were observed in our transcriptome analysis of HBx-Tg mice livers (Fig. S6A-B). By demonstrating a significant increase in overall m6A levels in HBV/HBx-expressing HCC cells (Fig. 3C), we found that this increase in m6A was specifically induced by HBx and displayed a dose-dependent relationship with HBx expression (Fig. S6C-D). Additionally, by assessing the transcription levels of the seven "writers", we observed notable increases in the mRNA and protein expression levels of methyltransferase-like 3 (METTL3), METTL14, and Wilms tumor 1-associating protein (WTAP) in HepG2.2.15 cells and HBx-expressing HepG2 cells in an HBx-dependent manner (Fig. 3D-E, Fig. S6E). These findings suggested that HBx promotes m6A levels mainly by upregulating METTL3, METTL14, and WTAP.

Analysis of the TCGA-LIHC cohort and the cross-linking and immunoprecipitation high-throughput sequencing (CLIP-seq) data reported previously 27 revealed that the METTL3, METTL14, and WTAP proteins potentially bind to *MALAT1*, which is linked to HCC pathogenesis (Fig.3F). MeRIP assays confirmed a significant increase in *MALAT1* m6A levels in HBV/HBx-expressing HCC cells (Fig. 3G). To investigate the involvement of METTL3, METTL14, and WTAP in regulating *MALAT1* m6A methylation, we constructed the corresponding Cas9 plasmids to knock down these "writers" (Fig. S6F). There was a significant decrease in *MALAT*1 m6A levels in HepG2.2.15 cells upon knockdown of METTL3 or METTL14 (Fig. 3H). We designed RNA pulldown biotinylated probes specific for *MALAT1* and confirmed their effectiveness (Fig. S6G), validating the direct binding between *MALAT1* and METTL3, METTL14, and WTAP in HepG2.2.15 cells and HBx-expressing HepG2 cells, with increased interactions in HBx-expressing cells (Fig. 3I). Moreover, subcellular fractionation in the RNA pulldown assays confirmed this binding regulation of HBV/HBx-associated *MALAT1* m6A methylation, specifically within the nucleus (Fig. 3J). Given that METTL3 serves as the primary catalytic enzyme in the methylation system, we generated both METTL3 wild-type (WT) and its mutant (Mut) enzyme active site to confirm its influence on *MALAT1* m6A regulation definitively, and MeRIP assays revealed a significant reduction in *MALAT1* m6A levels in the METTL3-Mut group compared with those in the METTL3-WT group (Fig. S6H).

*MALAT1* binding with METTL3, METTL14, and WTAP is regulated by its recognition of the m6A consensus motif with specific m6A sites. We utilized the lnCAR platform to analyze the secondary structure (maximum free energy was -2510 kCal/mol) of *MALAT1* (Fig. 3K). Based on the predicted structure and full length (F#2, 1-8779 nt) of *MALAT1*, eight smaller fragments were transcribed (F#3-10) via *in vitro* transcription. These fragments were biotinylated and subjected to RNA pulldown assays. We found that METTL3, METTL14, and WTAP were simultaneously pulled down by F#8 and F#9, which correspond to the 5490 nt to 7685 nt region of *MALAT1* (Fig. 3L), indicating that the potential regulatory domain for *MALAT1* m6A modification might reside within this flanking region. We subsequently employed the common prediction tools SRAMP and BERMP to screen the m6A-targeting sites in this domain further. The intersection of the predictions revealed two m6A sites: the 6021^A^ site in the eighth fragment (F#8) and the 7265^A^ site in the ninth fragment (F#9) (Fig. S6I). Additionally, the m6A binding motif of *MALAT1* was identified via the online platforms RMBase v2.0 and STAMP, which was consistent with 6021^A^ (GGACC) and 7265^A^ (GGACA) (Fig. S6J). To confirm our target screening results, dual-luciferase reporter gene plasmids based on pmirGLO that fused the linear sequence of the 5490 nt to 7685 nt fragment of *MALAT1* and the mutants of the 6021^C^ or/and 7265^C^ sites were constructed. Dual-luciferase reporter assays confirmed that both 6021^A^ and 7265^A^ were the m6A-targeting modification sites regulated by the binding of *MALAT1* to METTL3, METTL14, and WTAP in HepG2.2.15 cells (Fig. 3M). Moreover, site-directed mutagenesis plasmids with 6021^A^ or/and 7265^A^ sites based on pCDH-*MALAT1* were generated (Fig. S6K), and the results of MeRIP assays revealed that both 6021^A^ and 7265^A^ sites were necessary for the upregulation of *MALAT1* m6A modification in HepG2.2.15 cells (Fig. 3N). Additionally, the bacterial single-stranded RNase MazF assays identified the *MALAT1* 7265^A^ site with m6A methylation in HBV-related HepG2.2.15 cells (Fig. S6L). These findings suggested that the *MALAT1* m6A level was upregulated due to the recruitment of the METTL3/METTL14/WTAP methylation complex in HBV/HBx-related HCC cells, with specific binding at m6A sites identified as 6021^A^ and 7265^A^.

### IGF2BP3 interacts with and stabilizes* MALAT1* in a m6A-dependent manner

**Figure 4:**
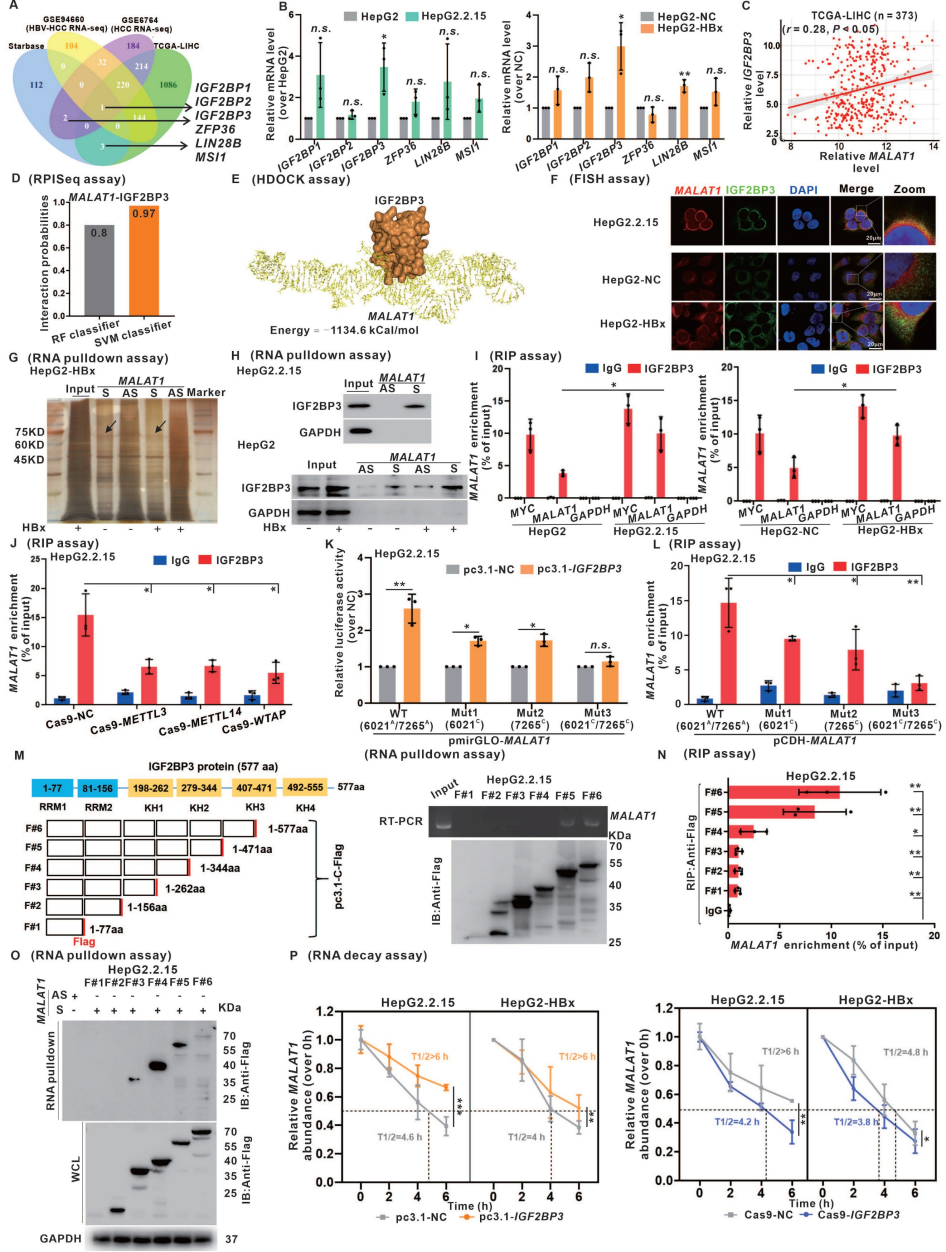
** IGF2BP3 interacts with and stabilizes *MALAT1* in a m6A-dependent manner. A** Venn diagram showing the overlap of DEGs from GSE6764, GSE94660, TCGA-LIHC cohort, and starBase database.** B** Relative expression of the six candidate RBPs was detected by qRT-PCR in HepG2.2.15 and HBx-expressing HepG2 cells. **C** Correlation analysis showing a positive relationship between IGF2BP3 and *MALAT1* in the TCGA-LIHC cohort. **D** Binding ability of *MALAT1* and IGF2BP3 was predicted by RPISeq. **E** Interaction between *MALAT1* and IGF2BP3 was predicted by HDOCK. **F** Representative images showing the colocalization of *MALAT1* (red) and IGF2BP3 (green) in HepG2.2.15 and HBx-expressing HepG2 cells. Scale bars: 20 μm. **G** RNA pulldown assays followed by silver staining of protein extracts from HBx-expressing HepG2 cells. **H** RNA pulldown assays were performed in HepG2.2.15 and HepG2 cells. **I** RIP assays were performed in HepG2.2.15 and HepG2 cells. **J** RIP assays were performed in HepG2.2.15 cells transfected with Cas9-*METTL3*, Cas9-*METTL14*, and Cas9-*WTAP* plasmids. **K** Dual-luciferase reporter assays were used to confirm the interaction between IGF2BP3 and two mutants (6021^C^ and 7265^C^) of the *MALAT1* m6A. **L** RIP assays were performed in HepG2.2.15 cells transfected with *MALAT1* containing the indicated mutations. **M** Scheme of Flag-tagged full-length IGF2BP3 (F#6) and the five truncated mutants (F#1: 1-77aa; F#2: 1-156aa; F#3: 1-262aa; F#4:1-344aa; F#5:1-471aa) were constructed (left). *In vitro* binding assays showing the enriched *MALAT1* in HepG2.2.15 cells detected by RT-PCR (right, upper panel) after incubation with full-length or truncated Flag-tagged IGF2BP3 protein validated by WB (right, lower panel). **N** RIP assays were performed in HepG2.2.15 cells transfected with plasmids containing the full-length or truncated constructs. **O** RNA pulldown assays were performed in HepG2.2.15 cells transfected with plasmids containing the indicated full-length or truncated constructs. **P** The RNA half-life of *MALAT1* was measured by performing RNA decay assaysin HepG2.2.15 and HBx-expressing HepG2 cells with IGF2BP3 overexpression or knockdown. *P < 0.05; **P < 0.01; ***P < 0.001. *n.s.*, not significant.

Growing evidence indicates that many lncRNAs can function to regulate the expression of target genes through direct interactions with proteins 28, 29. In the present study, we employed the starBase database to identify potential RNA-binding proteins (RBPs) that interact with *MALAT1*. By intersecting the results with those of the TCGA-LIHC and GEO HCC datasets, we identified six RBPs potentially interacting with *MALAT1* (Fig. 4A). Subsequent assessment in HBx-expressing cells highlighted significant upregulation of IGF2BP3 among the candidate RBPs in response to HBV/HBx expression (Fig. 4B). Furthermore, a significant positive correlation between IGF2BP3 and *MALAT1* expression in the TCGA-LIHC cohort was observed (Fig. 4C). Given these findings, we further investigated the role of IGF2BP3 in *MALAT1* regulation in HBV/HBx-related HCC. Immunohistochemistry analysis demonstrated that HBx expression significantly induced the upregulation of IGF2BP3 in the livers of HBx-Tg mice and was associated with HBV-related HCC (Fig. S7A-B). Pan-cancer analysis revealed elevated IGF2BP3 expression across various cancer types, particularly in liver tumors, compared with that in peritumor tissues (Fig. S7C). Additionally, Kaplan-Meier analysis revealed an association between high IGF2BP3 expression and lower OS and DFS in the TCGA-LIHC cohort (Fig. S7D). These findings highlight the potential role of IGF2BP3 regulation in HBV/HBx-related HCC prognosis and its potential association with *MALAT1*.

To investigate the interactive regulation between IGF2BP3 and* MALAT1*, we quantitatively assessed their binding potential via RPISeq (Fig. 4D) and established a molecular docking model via HDOCK, which illustrates their structural binding (Fig. 4E). Validation via RNA-FISH confirmed the IGF2BP3-*MALAT1* interaction in HBV/HBx-expressing HCC cells, with stronger binding observed in the presence of HBx (Fig. 4F). Subsequent RNA pulldown for silver staining (Fig. 4G) and WB (Fig. 4H) confirmed the presence of IGF2BP3 (~75 kDa) in the *MALAT1* fraction from HBx-expressing cells. In addition, RIP assays were utilized to validate the specific binding interaction between IGF2BP3 and* MALAT1*. Compared with the positive control *MYC*, which is known to bind with IGF2BP3 31, HBV/HBx expression increased the interactive binding of *MALAT1* with IGF2BP3 (Fig. 4I). An investigation of m6A-dependent regulation revealed that knockdown of METTL3, METTL14, or WTAP reduced the binding between IGF2BP3 and *MALAT1* (Fig. 4J). Dual-luciferase reporter assays using *MALAT1-*WT and its m6A site mutant plasmids in IGF2BP3-expressing HepG2.2.15 cells revealed that mutations of the 6021^C^ and 7265^C^ sites of *MALAT1* inhibited its binding activity with IGF2BP3 (Fig. 4K). Further RIP assays in cells transfected with *MALAT1* mutants demonstrated attenuated binding between IGF2BP3 and *MALAT1* upon mutation of these sites (Fig. 4L).

Considering the structure of IGF2BP3 protein (1-577 aa), which includes two RNA recognition motifs (RRMs) and four K homology (KH) domains, we constructed six flag-tagged recombinant plasmids containing overlapping truncated fragments (F#1-6) of IGF2BP3. In *in vitro* RNA binding assays, deletion of the KH3 domain (407-471 aa) of the flag-tagged IGF2BP3 protein significantly decreased, or even abolished, the binding interaction between IGF2BP3 and* MALAT1* (Fig. 4M-O). These results indicate the crucial role of the KH3 domain in the HBV/HBx-related IGF2BP3-targeting binding interaction with *MALAT1*.

To assess the regulatory effect of IGF2BP3 on *MALAT1* expression, our study revealed that *MALAT1* expression did not affect IGF2BP3 mRNA or protein levels in HBV/HBx-expressing HCC cells, regardless of whether *MALAT1* was overexpressed or knocked down (Fig. S7E-G). Conversely, in cells with IGF2BP3 overexpression or knockdown (Fig. S7H-I), overexpressing IGF2BP3 increased *MALAT1* levels, whereas knocking down IGF2BP3 decreased *MALAT1* expression in HBV/HBx-expressing HCC cells (Fig. S7J-K). Furthermore, RNA decay assays demonstrated that IGF2BP3 inhibited *MALAT1* decay rates, suggesting that IGF2BP3 stabilizes *MALAT1* in HBV/HBx-expressing HCC cells (Fig. 4P). These findings, along with the identification of IGF2BP3 as a newly characterized m6A lncRNA-binding protein and in conjunction with our earlier experiments shown in Fig. 3, suggest that IGF2BP3 might specifically interact with *MALAT1* to increase its stability in a m6A site-dependent manner in HBV/HBx-related HCC.

### IGF2BP3 promotes *MALAT1* nuclear-cytoplasmic shuttling in a m6A‑dependent manner

**Figure 5:**
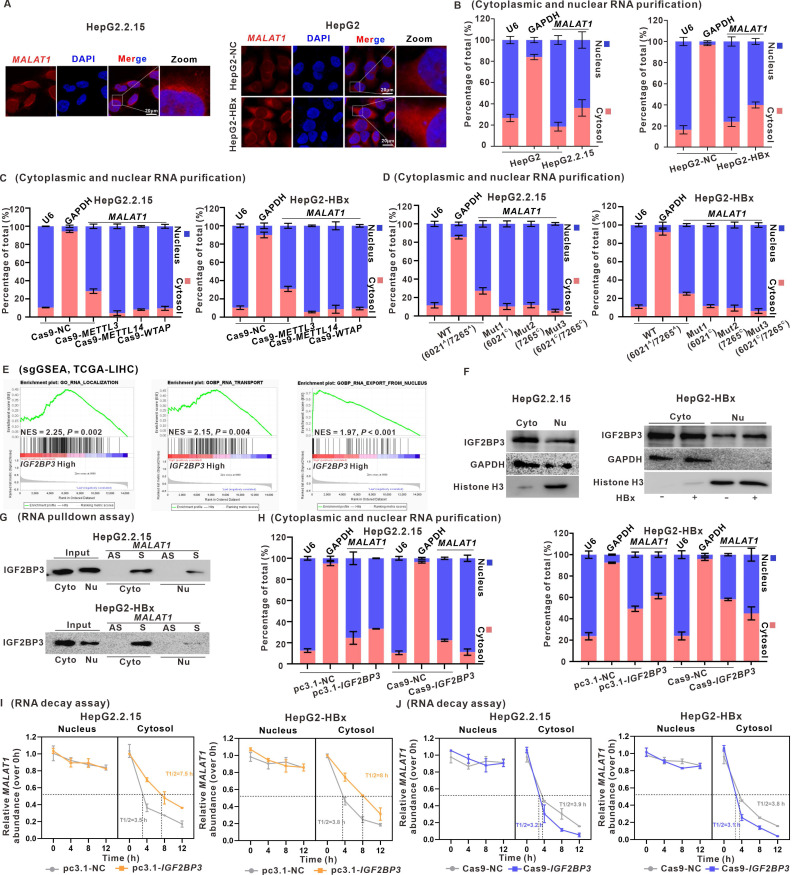
** IGF2BP3 promotes *MALAT1* nuclear-cytoplasmic shuttling in a m6A‑dependent manner.** HepG2.2.15 cells and HBx-expressing HepG2 cells were used for experiments, whereas HepG2 cells and HepG2-NC cells served as controls.** A** Representative images of FISH assays identifying the subcellular location of *MALAT1* (red) in the cells. Scale bars: 20 μm.** B** The subcellular distribution of *MALAT1* was analyzed via qRT-PCR in the cells. U6 and GAPDH were used as nuclear and cytoplasmic markers, respectively. **C-D** The subcellular distribution of *MALAT1* was analyzed via qRT-PCR in the cells transfected with the indicated plasmids. **E** sgGSEA of *IGF2BP3* in the TCGA-LIHC cohort. NES, normalized enrichment score. **F** The protein level of IGF2BP3 was detected by WB in the cytoplasmic and nuclear fractions of the cells.** G** RNA pulldown assays were conducted, and IGF2BP3 was pulled down by biotin-labeled sense *MALAT1* (S) but not by *MALAT1* antisense (AS) RNA in the cytoplasmic and nuclear fractions of the cells. **H** The subcellular distribution of *MALAT1* was analyzed via qRT-PCR in the cells transfected with the indicated plasmids.** I-J** RNA decay assays showing the effect of overexpressing and knocking down IGF2BP3 on *MALAT1* RNA half-life in the cells.

*MALAT1* is known to be highly abundant in the nucleus, specifically in nuclear speckles, which are nuclear compartments involved in pre-mRNA splicing and storage 32. Upon performing FISH and RNA nucleocytoplasmic isolation assays of *MALAT1,* we discovered the abundant presence of *MALAT1* in the cytoplasm in HBV/HBx-expressing HCC cells, challenging our original assumption that it was enriched primarily in the nucleus (Fig. 5A-B). Moreover, knockdown of METTL3, METTL14, or WTAP led to increased nuclear localization of *MALAT1* in HBV/HBx-expressing HCC cells, indicating a link between the nucleocytoplasmic shuttling of *MALAT1* and its m6A modification (Fig. 5C). Transfection of m6A site mutants (6021^C^ and/or 7265^C^) of *MALAT1* predominantly confined these overexpressed mutants to the nucleus of HBV/HBx-expressing HCC cells (Fig. 5D).

As shown in Fig. 4, we identified the role of IGF2BP3 in regulating the stability of *MALAT1* through m6A modification, which led us to consider whether IGF2BP3 participated in the regulatory nucleocytoplasmic shuttling of *MALAT1*. sgGSEA identified the involvement of IGF2BP3 in RNA localization (NES = 2.25), RNA transport (NES = 2.15), and RNA export from the nucleus (NES = 1.97) (Fig. 5E). The results of the cytoplasmic and nuclear protein separation assays demonstrated the presence of IGF2BP3 in the nucleus, whereas its levels were increased in HBV/HBx-expressing HCC cells (Fig. 5F). Furthermore, RNA pulldown experiments showed that IGF2BP3 binds to *MALAT1* in both the cytoplasm and nucleus (Fig. 5G). Moreover, IGF2BP3 knockdown resulted in the retention of *MALAT1* in the nucleus, whereas its overexpression facilitated its cytoplasmic distribution in HBV/HBx-expressing cells (Fig. 5H). As shown in Fig. 4P, IGF2BP3 enhanced *MALAT1* stability, prompting further investigation into whether this stability is linked to its cellular localization. Following the separation of cytoplasmic and nuclear RNA, RNA decay assays revealed that HBx primarily prevented *MALAT1* degradation in the cytoplasm, with minimal degradation in the nucleus (Fig. S8A-B), possibly due to *MALAT1* binding to multiple RBPs in the nucleolus. Additionally, IGF2BP3 also reduced *MALAT1* decay rates in the cytoplasm (Fig. 5I-J). These findings indicate that IGF2BP3, which binds to m6A-modified *MALAT1*, facilitates the nuclear export of m6A-modified *MALAT1* for its cytoplasmic localization and RNA stabilization.

### Targeting *MALAT1 in vivo* with ASO-*MALAT1* treatment effectively suppresses xenograft tumor progression in HBx-related HCC

**Figure 6:**
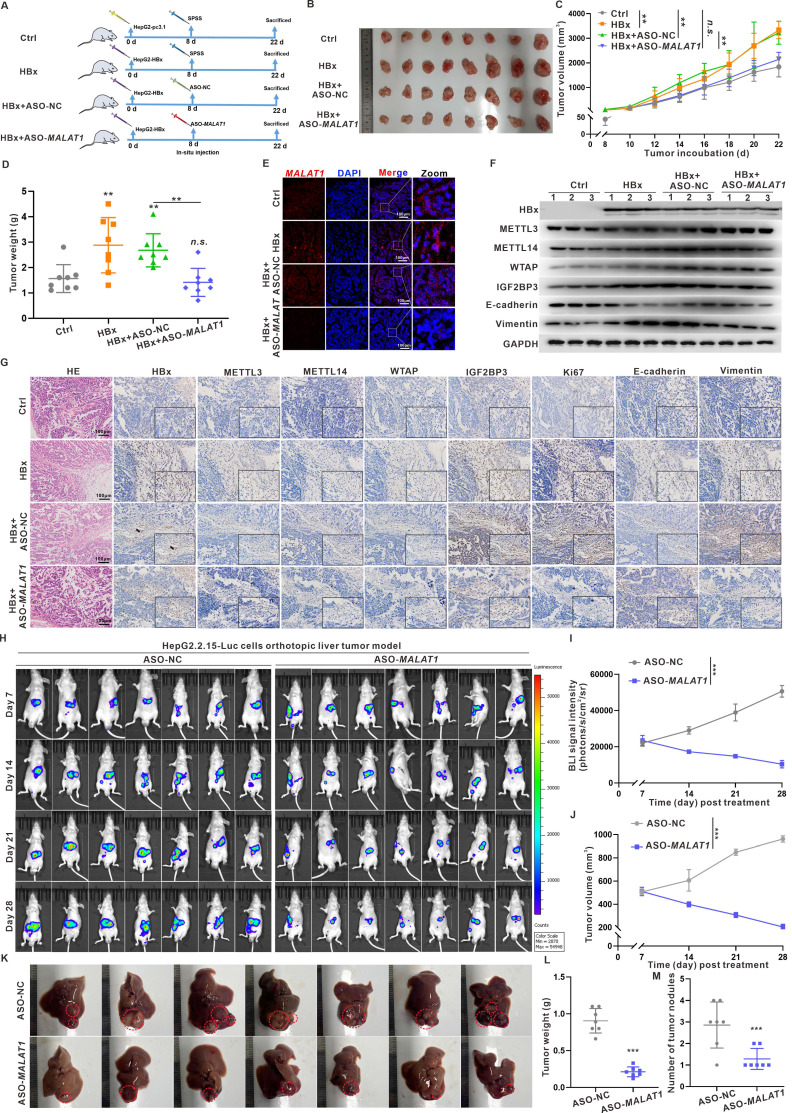
** Targeting *MALAT1 in vivo* with ASO-*MALAT1* treatment effectively suppresses xenograft tumor progression in HBx-related HCC. A-G** BALB/c nude mice were used to construct the subcutaneous xenograft models with HBx-expressing HepG2 cells. The mice were divided into four groups: Ctrl (HepG2-pc3.1-NC), HBx (HepG2-pc3.1-HBx), HBx+ASO-NC, and HBx+ASO-*MALAT1* (n = 8 per group). For *MALAT1-*targeting intervention, mice were treated with ASO-*MALAT1*(5 nmol per injection, once every 2 days, for a total of 7 injections), while control oligonucleotides (ASO-NC) served as a negative control. **A** Schematic diagram of the experimental design, where SPSS represents stroke-physiological saline solution. **B** Representative images showing the subcutaneous tumor xenografts. **C** Tumor weights of the xenografts were evaluated. **D** Tumor growth curves of the xenografts were plotted. **E** Representative images showing FISH assays of the RNA levels of *MALAT1* (red) in the xenograft tumors. Nuclei were counterstained with DAPI (blue). Scale bars: 100 μm. **F** Levels of HBx, METTL3, METTL14, WTAP, IGF2BP3, E-cadherin, and Vimentin proteins in the xenograft tumors were detected by WB. **G** Representative images showing the HE staining, HBx, METTL3, METTL14, WTAP, IGF2BP3, Ki67, E-cadherin, and Vimentin immunostaining of the xenograft tumors. Scale bars: 100 μm. **H-M** An orthotopic liver tumor model was established in nude mice by injecting HepG2.2.15-Luc cells. Mice were then treated with ASO-*MALAT1*, while ASO-NC were used as a negative control (n = 7 per group). **H** Representative bioluminescence images of orthotopic liver tumors, with imaging performed on days 7, 14, 21, and 28 post-treatment. **I-J** Quantification of tumor burden based on total bioluminescence signal intensity **(I)** and tumor volume **(J)** over time. **K** Gross images of excised livers from each group on day 28. Intrahepatic orthotopic tumor nodules are outlined with dashed circles. **L-M** Quantification of tumor weight **(L)** and number of intrahepatic tumor nodules **(M)** is presented in dot plots. *P < 0.05; **P < 0.01; ***P < 0.001.* n.s.*, not significant.

To assess the effects of *MALAT1* and its m6A methylation regulatory signaling axis on HBx-related HCC migration and metastasis, a nude mouse model harboring subcutaneous xenograft liver tumors was used (Fig. 6A). There were no observable changes in the body weights of the mice in the various groups (Fig. S9A). Compared with control tumors, xenograft tumors derived from HBx-expressing HepG2 cells were larger in size, heavier in weight, and grew faster (Fig. 6B-D). When *MALAT1* was knocked down with ASO-*MALAT1* in xenograft tumors derived from HBx-expressing HepG2 cells*,* significantly smaller tumor sizes, weights, and volumes were detected than in the HBx+ASO-NC group (Fig. 6B-D). The *MALAT1* level and its distribution in xenograft tumor tissues were increased in the HBx group (Fig. 6E, Fig. S9B), whereas they were decreased in the ASO-*MALAT1* group ((Fig. 6E, Fig. S9B). Moreover, the overall m6A level and its related METTL3, METTL14, and WTAP protein levels, as well as cytoplasmic *MALAT1*-exporting IGF2BP3 and its distribution in xenograft tumor tissues, were increased in the HBx group (Fig. 6F-G, Fig. S9C). During the malignant progression of HBx-related HCC, compared with those in the HBx group, the levels of HBx-induced Ki67 and Vimentin in xenograft tumor tissues were lower in the ASO-*MALAT1* group (Fig. 6F-G), indicating that *MALAT1* modulates HBx-driven tumor growth and metastatic potential through an m6A-dependent mechanism.

To further validate the metastatic function of *MALAT1* in a physiologically relevant model, we established an orthotopic liver tumor model using luciferase-labeled HepG2.2.15 (HepG2.2.15-Luc) cells. There were no observable changes in the body weights of the mice in the various groups (Fig. S9D). Mice receiving ASO-*MALAT1* treatment showed significantly reduced bioluminescent signal intensity over 28 days, suggesting suppressed tumor progression (Fig. 6H-J). At necropsy, tumors in the ASO-*MALAT1* group were visibly smaller with fewer intrahepatic nodules, indicating local tumor suppression. Moreover, qRT-PCR analysis confirmed that ASO-*MALAT1* treatment markedly reduced hepatic *MALAT1* expression levels (Fig. S9E). Subsequent WB revealed increased E-cadherin and decreased Vimentin expression, indicating suppression of EMT following *MALAT1* knockdown (Fig. S9F). Although no lung metastases were observed—likely due to the relatively short duration of the experiment—abdominal dissemination was detected in control animals but was notably reduced in the ASO-*MALAT1* group (Fig. 6K-M). These observations provide functional evidence that *MALAT1* contributes to both tumor growth and early metastatic dissemination in HBV-related HCC. Together, findings from the xenograft and orthotopic models underscore the critical role of *MALAT1* in promoting HBx-mediated hepatocarcinogenesis and metastasis. Suppression of *MALAT1* not only attenuates tumor proliferation but also limits intrahepatic expansion and peritoneal spread, supporting its therapeutic potential as a dual-target regulator of tumor progression and early dissemination.

### Anti-HBx gene delivery via transposons suppresses *MALAT1*-m6A-initiated HBV-related hepatocarcinogenesis *in vivo*

**Figure 7:**
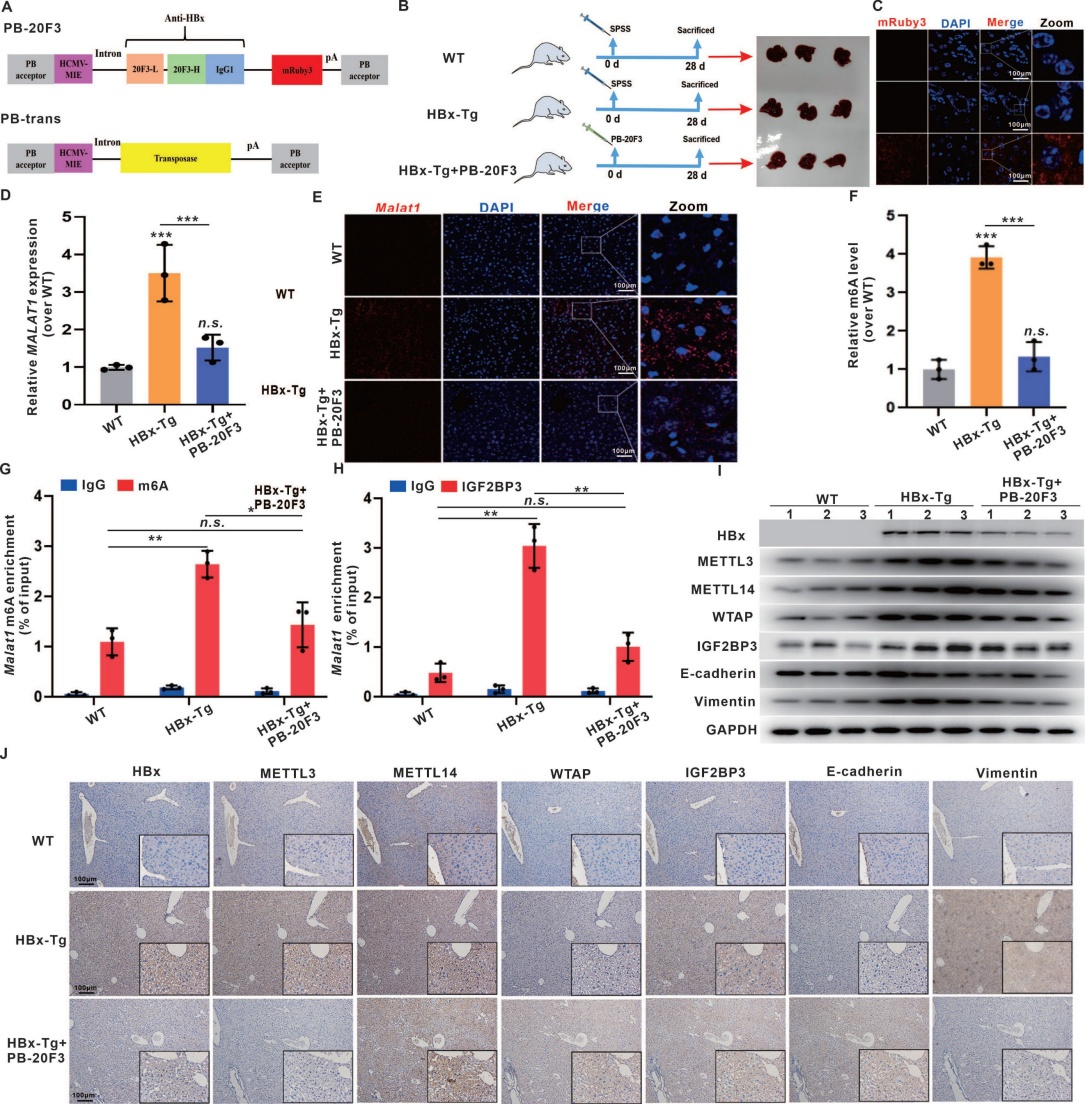
** Anti-HBx gene delivery via transposons suppresses *MALAT1*-m6A-initiated HBV-related hepatocarcinogenesis *in vivo*.** HBx-Tg mice were injected with 0.3 mL of TransIT-EE containing PB-20F3 (10 μg) and PB-Trans (5 μg) once every 4 days for a total of 7 injections. **A** Scheme of the plasmids PB-20F3, with SPSS used as a negative control. **B** Schematic diagram of the experimental design for HBx-targeting intervention in HBx-Tg mice. **C** Representative images showing fluorescence of mRuby3 in livers from the different groups, with nuclei counterstained with DAPI (blue). Scale bars: 100 μm. **D** The relative expressions of *MALAT1* in the livers was detected by qRT-PCR. **E** Representative images showing FISH assays of the RNA levels of *MALAT1* (red) in the livers, with nuclei counterstained with DAPI (blue). Scale bars: 100 μm. **F** The overall m6A content in the livers was analyzed by m6A quantitation analysis. **G** MeRIP assays were performed in the livers, while the abundance of *MALAT1* with anti-m6A antibodies was measured by qRT-PCR and normalized to that of IgG. **H** RIP assays were performed with IGF2BP3 antibody in the livers, while the abundance of *MALAT1* was measured by qRT-PCR. **I** The levels of HBx, METTL3, METTL14, WTAP, IGF2BP3, E-cadherin, and Vimentin proteins in the livers were detected by WB.** J** Representative images showing HBx, METTL3, METTL14, WTAP, IGF2BP3, E-cadherin, and Vimentin immunostaining of livers. Scale bars: 100 μm. *P < 0.05; **P < 0.01; ***P < 0.001. *n.s.*, not significant.

Given the pivotal role of HBx in HBV-related hepatocarcinogenesis initiation and progression, HBx-targeting suppression represents a promising strategy to inhibit HBV replication and prevent HBV-related HCC 17, 20. In previous studies, we successfully suppressed the biofunction of intercellular HBx expression in HCC cells via an anti-HBx monoclonal antibody (mAb) delivered through a recombinant anti-HBx plasmid (pTT5-anti-HBx) 20. In this study, we further expanded our anti-HBx intervention to target the *MALAT1*-m6A-initiated pathway, thereby suppressing hepatocarcinogenesis phenotypes in HBx-Tg mice. We employed the PiggyBac system, a mammalian gene transfer system based on a transposon, to express the anti-HBx mAb and interfere with HBx expression in mice (Fig. 7A-B). Concurrently, hydrodynamic-based gene delivery (HGD) was utilized as a highly efficient technique to introduce the anti-HBx plasmid into murine hepatocytes 33. HBx-Tg mice were administered an intravenous injection of a PB-20F3 transposon containing an anti-HBx mAb-encoding sequence (Fig. 7A-B), which was then delivered into the mouse liver via HGD to efficiently express the anti-HBx mAb, visualized with mRuby3 (Fig. 7C).

Although the HBx-Tg mice did not develop overt liver tumors during the 6-month observation period, consistent with previous reports requiring a longer latency (14-18 months) for spontaneous HCC development 34, 35, histopathological examination revealed clear preneoplastic alterations. HE staining identified hepatocellular nuclear atypia (e.g., anisokaryosis, hyperchromasia, prominent nucleoli), while immunohistochemistry showed elevated expression of early HCC markers—GPC3, AFP, Ki67, and PCNA—and activation of EMT-associated markers (Fig. S10). These findings demonstrate that persistent HBx expression initiates early oncogenic events prior to macroscopic tumor formation.

Importantly, anti-HBx treatment via the PB-20F3 plasmid significantly reversed these molecular and histological alterations. The expression and hepatic distribution of *Malat1* were elevated in HBx-Tg mice livers but reduced in the PB-20F3 group (Fig. 7D-E). The overall m6A level and related METTL3, METTL14, and WTAP protein levels, as well as their distribution in liver tissues, were increased in the HBx group (Fig. 7F-J), whereas these HBx-induced m6A regulators were downregulated in the PB-20F3 group (Fig. 7F-J). In particular,* Malat1* m6A enrichment and *Malat1* binding with IGF2BP3 were increased in the HBx group but downregulated in the PB-20F3 group (Fig. 7G-H). Compared with those in the HBx group, the levels of HBx-induced Vimentin in liver tissues were lower in the PB-20F3 group (Fig. 7I-J). Collectively, these data demonstrate that genetic intervention targeting HBx not only mitigates *MALAT1* overexpression but also disrupts the associated m6A epitranscriptomic network that contributes to early hepatocarcinogenic signaling.

## Discussion

**Figure 8:**
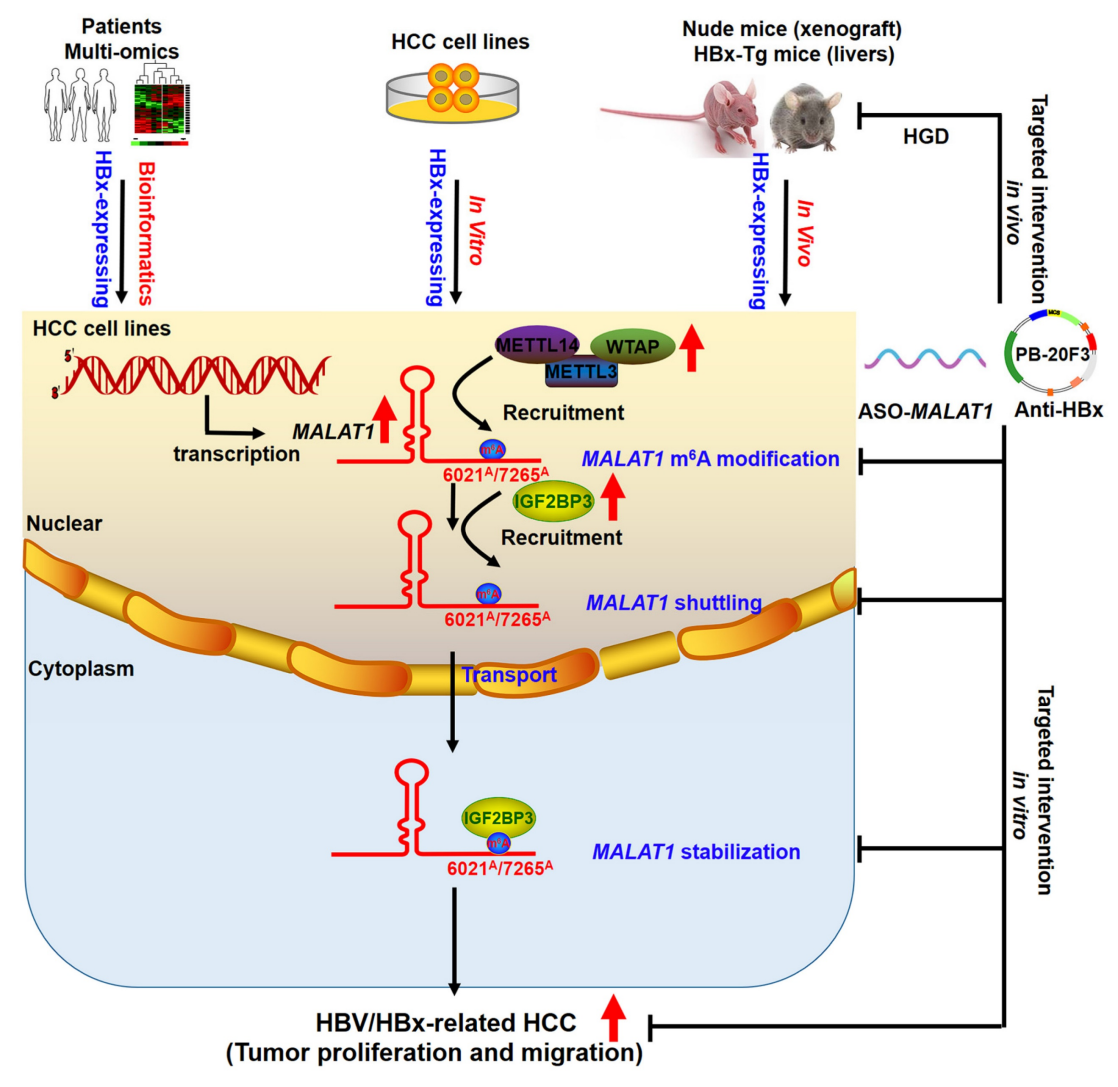
Schematic diagram illustrating the mechanisms of IGF2BP3-mediated m6A modification of *MALAT1* in promoting the malignant progression of HBV/HBx-related HCC, as well as potential strategies for epigenetic or genetic intervention.

HBx acts as an oncoprotein and plays a key role in HBV-related HCC by influencing epigenetic modifications and genetic regulation. Epigenetic alterations, including DNA methylation, histone acetylation, and microRNA (miRNA) regulation, have been reported as early events in tumorigenesis, contributing to the malignant transformation of hepatocytes 36. Emerging evidence has implicated lncRNAs in HBV-induced carcinogenesis via interactions with epigenetic modification complexes. For example, HBx repressed LINC01431 transcription and decreased the stability of PRMT1, resulting in reduced PRMT1 enrichment on cccDNA and increased cccDNA accessibility and transcription 37. Despite these findings, the precise role and mechanisms of lncRNAs in HBV-related HCC, particularly how HBx promotes carcinogenesis and tumorigenesis through lncRNAs, remain unclear and warrant further investigation. This study comprehensively analyzed differentially expressed lncRNAs in HBV-related HCC patients via multiple datasets and validated their significance in the liver transcriptomes of HBx-Tg mice. Our investigation revealed that HBx expression alters the lncRNA profile in HBx-expressing cells, indicating that a distinct lncRNA profile is associated with HBV-related HCC tumorigenesis. Notably, *MALAT1* was significantly overexpressed in response to HBx across multiple datasets and in HBx-Tg mice, and this overexpression was correlated with poor prognosis in HBV-related HCC patients. *MALAT1,* an oncogenic lncRNA, promotes cancer progression through various mechanisms, such as miRNA sequestration, autophagy stimulation, and the induction of epithelial-mesenchymal transition 32. However, the specific involvement of HBx in elevated *MALAT1* levels in HCC and the underlying regulatory mechanisms have remained elusive. Our results revealed that HBx induced *MALAT1* upregulation, which is correlated with poor prognosis in HBV-related HCC patients. Additionally, the knockdown of *MALAT1* in HBx-expressing cells suppressed hepatocarcinogenesis phenotypes, and *in vivo* experiments demonstrated the inhibition of xenograft tumor growth upon *MALAT1* knockdown (Fig. 8). These findings emphasize the significant role of *MALAT1* in HBV-related HCC progression and prognosis, indicating that *MALAT1* is a potential therapeutic target.

m6A modification governs various cellular and viral RNA functions 38-40. Viral infections can induce cellular lncRNAs with antiviral effects, while viruses exploit lncRNAs to regulate metabolic networks for their survival 41. HBx interacts with METTL3/14 enzymes, recruiting them to both viral cccDNA and host chromosomal loci, indicating that m6A is involved in HBx-driven hepatocarcinogenesis 42. Mechanistically, our study revealed that HBx modulates the m6A epitranscriptomic landscape to enhance *MALAT1* stability. HBx upregulated METTL3, METTL14, and WTAP expression, increasing m6A deposition on *MALAT1*, particularly at the 6021^A^ and 7265^A^ sites. MeRIP and pulldown assays confirmed *MALAT1* binding to these m6A writers. IGF2BP3, a known m6A reader, was found to directly interact with and stabilize m6A-modified *MALAT1* via its KH3 domain, as validated by RIP and RNA decay assays (Fig. 8). These findings reveal a novel HBx-mediated epigenetic regulatory axis involving the METTL3-m6A-*MALAT1*-IGF2BP3 cascade in HBV-related liver tumorigenesis.

Identifying targeted interventions for the specific functions of HBx presents a challenge. Given the crucial role of HBx in HBV replication and cell survival, targeting HBx is essential for preventing HBV-associated liver disease. Monoclonal antibodies have emerged as therapeutic options for infectious diseases and cancer. Previous studies have demonstrated that anti-HBx antibodies can reduce intracellular HBx levels through TRIM21-mediated protein degradation, while also activating the NF-κB, AP-1, and IFN-β pathways, leading to enhanced antiviral responses in host cells 43. In our study, we utilized a hydrodynamic delivery system to express an intracellular anti-HBx mAb (PB-20F3) in HBx-Tg mice. Although this model did not develop macroscopic tumors within six months, consistent with prior findings, histological analysis revealed marked preneoplastic changes in HBx-Tg livers, including nuclear atypia, elevated GPC3, AFP, Ki67, and activation of EMT markers. Anti-HBx treatment effectively suppressed these changes, restored tissue architecture, and downregulated *MALAT1*, METTL3/14, and IGF2BP3 expression (Fig. 8). These findings demonstrate that anti-HBx intervention attenuates early oncogenic signaling and epigenetic remodeling driven by HBx.

Altogether, our data establish that HBx promotes hepatocarcinogenesis via *MALAT1* upregulation and m6A-mediated stabilization, thereby facilitating tumor growth and metastasis. The orthotopic liver model, transcriptomic analysis, and HBx-Tg mouse interventions collectively elucidate the role of the HBx/*MALAT1*/IGF2BP3 axis in HBV-related HCC (Fig. 8). These findings provide mechanistic insights and experimental evidence supporting lncRNA-based therapeutic strategies in HBV-driven liver cancer, with *MALAT1* serving as both a prognostic biomarker and a potential therapeutic target.

## Supplementary Material

Supplementary methods, figures and tables.

## Abbreviations

HBV: Hepatitis B virus
HBx: Hepatitis B virus X protein
HCC: Hepatocellular carcinoma
lncRNA: Long non-coding RNA
*MALAT1*: Metastasis-associated lung adenocarcinoma transcript 1
m6A: N6-methyladenosine
IGF2BP3: Insulin like growth factor 2 mRNA-binding protein 3
ASO: antisense oligonucleotides
HGD: Hydrodynamics-based gene delivery
HBx-Tg: HBx transgenic
ceRNAs: Competing endogenous RNAs
cccDNA: Covalently closed circular DNA
FISH: Fluorescence in situ hybridization
WB: Western blotting
IHC: immunohistochemistry
qRT-PCR: Quantitative real-time PCR
H&E: Hematoxylin and eosin
NC: Negative control
BLI: Bioluminescence imaging
WT: Wild-type
DEGs: Differentially expressed genes
OS: Overall survival
DFS: Disease-free survival
sgGSEA: Single-gene gene-set enrichment analysis
ALD: Alcoholic liver disease
EMT: Epithelial to mesenchymal transition
rcccDNA: Relaxed circular cccDNA
METTL3/14: Methyltransferase-like 3/14
WTAP: Wilms tumor 1-associating protein
CLIP-seq: Cross-linking and immunoprecipitation high-throughput sequencing
RBP: RNA-binding protein
RRMs: RNA recognition motifs
mAb: Monoclonal antibody
