# Supplementary material for: MALAT1 promotes malignancy of HBV-related hepatocellular carcinoma by regulating IGF2BP3-mediated nuclear-cytoplasmic shuttling
Source: Int J Biol Sci. 2025 Jul 28;21(11):4942–60. doi: 10.7150/ijbs.112133 (PMC12374813; doi:10.7150/ijbs.112133)
Supplement: Supplementary file 1 — Supplementary methods, figures and tables. [file ijbsv21p4942s1.pdf]

Supplementary materials

MALAT1 promotes malignancy of HBV-related hepatocellular carcinoma by regulating IGF2BP3-mediated nuclear-cytoplasmic shuttling

Ze-Bang Du[1†], Xin-Mou Wu[1†], Tun Han[1†], Yu-Xin Cai[1], Bo Qian[1], Yu-Shi Shen[1], Han-Yu Zhang[1], Jia-Shen Wu[1], Jie He[1], Xiao-Xuan Chen[1], Dong-Bei Guo[1], Hang-Tian Zhong[2], Xiong Li[2], Lei Zhang[1], Xiao-Ming Luo[2*], Wen-Gang Li[3*], Yu-Chun Lin[1*], and Zhong-Ning Lin[1*]

[1] State Key Laboratory of Vaccines for Infectious Diseases, Xiang An Biomedicine Laboratory, Xiang'an Hospital of Xiamen University, National Innovation Platform for Industry-Education Integration in Vaccine Research, School of Public Health, Xiamen University, Xiamen 361102, China

[2] Department of Preventive Medicine, School of Public Health, Chengdu Medical College, Chengdu, 610500, China.

[3] Department of Hepatobiliary Surgery, Cancer Research Center, Xiang'an Hospital of Xiamen University, School of Medicine, Xiamen University, Xiamen, Fujian 361102, China.

[†] These authors contributed equally to this work.

[*] Corresponding author: lxm@cmc.edu.cn (X.-M. Luo); lwgang@xmu.edu.cn (W.-G. Li); linych@xmu.edu.cn (Y.-C. Lin); and linzhn@xmu.edu.cn (Z.-N. Lin)

Tel: +86 592 2880615; Fax: +86 592 2881578.

Supplementary materials and methods

Gene Expression Omnibus (GEO) and the Cancer Genome Atlas (TCGA) data analysis

For GEO analysis, publicly available RNA expression datasets were retrieved from the GEO database to investigate lncRNA alterations across etiologically distinct types of HCC. Datasets were systematically screened to include HBV-, HCV-, alcohol-related liver disease (ALD)-, and non-alcoholic fatty liver disease (NAFLD)-associated HCC, as well as matched non-tumor liver tissues when available. Detailed information is presented in Supplementary Table S1. For TCGA analysis, RNA sequencing data and clinical information of HCC samples (n = 373), as well as RNA sequencing data of 50 adjacent non-cancerous samples, were obtained from Xena database. Standardization and expression value calculation were performed on the original datasets using Limma packages in R software (version: x64 4.0.2). The absolute log2 fold change (log2FC) ≥ 1.2 and $P < 0.05$ were considered statistically significant and used to screen differentially expressed genes (DEGs). Volcano plots and heatmaps were generated based on the obtained DEGs. Gene ontology (GO) and Kyoto Encyclopedia of Genes and Genomes (KEGG) pathway enrichment analyses were performed to identify pathways significantly affected by the hepatocarcinogenesis process of DEGs. Single-gene gene set enrichment analysis (sgGSEA) was used to determine various signaling signatures related to the expression of *MALAT1* and *IGF2BP3* genes, and pathways with a false discovery rate (FDR) < 0.25 were considered significantly enriched.

RNA sequencing analysis

RNA sequencing was conducted by Genedenovo Biotech Co., Ltd (Guangzhou, Guangdong, China) following established protocols. HepG2.2.15 cells treated with ASO-

MALAT1 and three biological replicates from the ASO-NC control group were subjected to transcriptomic analysis. DEGs were identified using criteria of $|\log 2FC| \geq 1$ and $P < 0.05$. GO, KEGG, and GSEA pathway enrichment analyses were subsequently conducted to elucidate enriched pathways associated with tumor metastasis and proliferation.

Cell lines and culture conditions

Human HCC cell lines (Hep3B, BEL-7404, BEL-7402, HepG2, HuH7, PLC/PRF/5, MHCC-97H, and HepG2.2.15) and human normal liver cells (L02) were maintained in our laboratory. HepG2.2.15 cells were derived from HepG2 cells with stable transfection HBV genome expression [1]. All cells were cultured in Dulbecco's Modified Eagle's Medium (DMEM) (Gibco; Waltham, MA, USA) supplemented with 10% fetal bovine serum (FBS) and 1% (v/v) penicillin-streptomycin at 37 °C in a humidified atmosphere containing 5% CO_2 (Thermo; Waltham, MA, USA).

Plasmid construction and antisense oligonucleotides (ASOs) for cell transfection

Based on the lentiCRISPR v2 plasmid system (Addgene; Watertown, MA, USA), we constructed recombinant plasmids Cas9-*MALAT1*, -*METTL3*, -*METTL14*, -*WTAP*, and -*IGF2BP3* to knockdown the expression of *MALAT1*, METTL3, METTL14, WTAP, and IGF2BP3, respectively. The construction process can refer to our previous research [2]. Briefly, small guide RNA (sgRNA) targeting *MALAT1*, *METTL3*, *METTL14*, *WTAP*, and *IGF2BP3* genes were designed, annealed, and cloned into the LentiCRISPRv2 vector. The dead CRISPR/Cas9-based Synergistic Activation Mediator system (dCas9-SAM) (Addgene) was used to achieve stable *MALAT1* overexpression. dCas9-*MALAT1* was constructed by standard procedures, including enzyme digestions, PCR, and subcloning, according to previous study [3,

4].

For overexpression of IGF2BP3, the full-length complementary human *IGF2BP3* cDNA was amplified and cloned into the expression vector to construct the recombinant plasmid pc3.1-*IGF2BP3*. Plasmids pc3.1-HBV, pc3.1-HBx, and HBV-x-null plasmids were maintained in our laboratory. Flag-tagged expression plasmids encoding HBp, HBs, HBc, and HBx were obtained from Miaolingbio (Wuhan, Hubei, China). ASOs, synthesized by RiboBio (Guangzhou, Guangdong, China), were employed in a mixed-modality strategy combining ASOs and RNAi reagents to enhance knockdown efficacy [5], given *MALAT1*'s localization in both nuclear and cytoplasmic compartments. Transfection of cells with ASOs was performed using Lipofectamine 2000 (Invitrogen; Carlsbad, CA, USA) following the manufacturer's instructions. The primers for various plasmid constructions are listed in Supplementary Table S2.

Quantitative real-time polymerase chain reaction (qRT-PCR) assay

Total RNA was isolated using TRIzol reagent (TaKaRa; Kyoto, Japan) according to the product description. Cytoplasmic and nuclear RNA was extracted using an RNA Purification Kit (Norgen Biotek; Thorold, ON, Canada). For lncRNA and mRNA, reverse transcription was performed using the PrimeScript™ RT Master Mix (TaKaRa). qRT-PCR was performed with SYBR® Premix ExTaq II kit (TaKaRa) using a CFX96 Touch™ Detection System (Bio-Rad, Hercules, CA, USA). The relative expression levels of the indicated lncRNA and mRNA were calculated via the $2^{-\Delta\Delta Ct}$ method and normalized to endogenous *GAPDH* or U6. The primers for qRT-PCR assay are listed in Supplementary Table S3.

Western blotting (WB)

WB analysis was performed as described previously [1]. Detailed information on the primary antibodies, including the manufacturers, catalog numbers, dilutions used in WB, RIP, and IHC, molecular weights, and species reactivity, is listed in Supplementary Table S4.

Quantification of HBsAg and HBeAg by ELISA

The concentrations of hepatitis B surface antigen (HBsAg) and hepatitis B e antigen (HBeAg) in the culture supernatants of HepG2.2.15 cells were determined using commercially available enzyme-linked immunosorbent assay (ELISA) kits (Kehua Biotech; Shanghai, China), following the manufacturer's instructions. Absorbance was measured at 450 nm using a microplate reader, and antigen levels were quantified based on standard curves provided by the kit.

Extraction and analysis of pgRNA, intracellular HBV DNA, and rcccDNA

The method for the extraction and quantification of pregenomic RNA (pgRNA), intracellular HBV DNA, and relaxed circular cccDNA (rcccDNA) was adapted from previous studies [6, 7]. Briefly, total RNA and DNA were extracted from HepG2.2.15 cells using TRIzol (TaKaRa) reagent and the QIAamp DNA Mini Kit (Qiagen; Hilden, Germany), respectively, according to the manufacturers' protocols. For pgRNA detection, total RNA was treated with DNase I (Takara), reverse transcribed using the PrimeScript RT Reagent Kit (Takara), and quantified by qPCR using primers specific to the pgRNA region. Intracellular total HBV DNA and rcccDNA were measured via qPCR after treatment with plasmid-safe DNase (Epicentre; Madison, WI, USA) to selectively degrade linear DNA and enrich rcccDNA. Quantitative analysis was performed using SYBR Green Master Mix (Takara) on a LightCycler 480 system

(Roche; Basel, Switzerland). *GAPDH* was used as an internal control for normalization. Primer sequences are listed in Supplementary Table S3.

Immunohistochemistry (IHC) analysis and HE staining

Tissue sections from tumor samples of HCC patients, livers of HBx-Tg mice, and xenograft tumors of BALB/c nude mice were fixed with 4% paraformaldehyde (PFA), embedded in paraffin, and serially sectioned at 4 μm thickness. For IHC analysis, the sections were processed using an UltraSensitive™ SP IHC Kit (MXB; Fuzhou, Fujian, China) as described in our previous study [8]. Information on the antibodies, including those against METTL3, METTL14, WTAP, IGF2BP3, E-cadherin, Vimentin, GPC3, AFP, PCNA, and Ki67, is listed in Supplementary Table S4. For H&E staining, the slides were stained with hematoxylin and eosin (Beyotime; Shanghai, China) and dehydrated through increasing concentrations of ethanol and xylene.

Immunofluorescence (IF) assay

IF analysis was performed as described previously [1]. Information on the antibodies, including those against IGF2BP3, is listed in Supplementary Table S4.

Fluorescence *in situ* hybridization (FISH)

Cy3-labeled *MALAT1* probes were synthesized by GenePharma (Shanghai, China). The oligonucleotide sequences are available in Supplementary Table S5. FISH analyses of tissue serial sections and cell-mounted slides were performed with an RNA FISH SA-Biotin kit (GenePharma) according to the manufacturer's instructions. The signals were measured using a fluorescence confocal microscope (Zeiss LSM 880; Oberkochen, Baden-Württemberg,

Germany).

Dual-luciferase reporter assay

For the *MALAT1* m6A sites luciferase reporter assays, sequences containing wild-type (WT) bases 5490 nt to 7685 nt of *MALAT1* or mutated m6A sites (6021^{C} and 7265^{C}) were amplified and cloned into the pmirGLO firefly luciferase reporter (Promega; Madison, WI, USA). Dual-luciferase reporter assays were performed following the established protocol. Briefly, HepG2.2.15 and HepG2 cells were seeded at 3×10^{4} cells per well in 24-well plates. After 24 h, co-transfection was conducted with 0.1 μg/well of pmirGLO plasmid containing Renilla luciferase for internal normalization and the recombinant plasmids containing *MALAT1* m6A sites 6021^{A} and 7265^{A} or their mutations 6021^{C} and 7265^{C}.

RNA pulldown assay

PCR primers containing the T7 promoter were synthesized, and the DNA template was obtained by PCR amplification of pCDH-*MALAT1* (Addgene). *In vitro* transcription was performed using a Ribo™ RNAmax-T7 Biotin-Labeled Transcription Kit (RiboBio). The primers are listed in Supplementary Table S5. The RNA pulldown assay was performed using the RNA Pulldown Kit (BersinBio; Guangzhou, Guangdong, China) according to the manufacturer's instructions. HepG2.2.15 and HepG2 cells expressing HBx were lysed and incubated with biotin-labelled *MALAT1* probes on a shaker overnight. The mixed RNA-protein binding reaction solution was incubated with magnetic beads for 1 h. The elution of RNA-binding protein complexes was executed using elution buffer at 50 °C for 40 min. The eluate was collected, and the proteins bound to *MALAT1* were detected by silver staining and WB.

RNA immunoprecipitation (RIP) assay

RIP experiments were conducted using the RIP Kit (BersinBio) following the provided guidelines. Specifically, 2×10^7 HepG2.2.15 and HepG2 cells expressing HBx were lysed with RIP lysis buffer and then incubated with magnetic beads conjugated with primary antibodies. Antibodies targeting IGF2BP3 and immunoglobulin G (IgG) are detailed in Supplementary Table S4. The immunoprecipitated *MALAT1*-containing RNA was reverse transcribed and directly examined using qRT-PCR, following the established protocols for RNA extraction, cDNA synthesis, and qRT-PCR.

Methylated RNA immunoprecipitation (MeRIP) assay

MeRIP was conducted using the MeRIP Kit (BersinBio) following the manufacturer's protocol and utilizing an anti-m6A antibody (Abcam; Cambridge, MA, USA). Initially, RNA isolation was performed, followed by fragmentation using RNA fragmentation buffer. A fraction of the total RNA was reserved as input, while the rest underwent MeRIP with m6A antibody-coated magnetic beads A/G. Subsequently, the complexes containing *MALAT1* were thoroughly washed, eluted, purified, and examined via qRT-PCR, following the established procedures for RNA extraction, cDNA synthesis, and qRT-PCR.

Overall RNA m6A quantification

The overall m6A levels in total RNA were assessed using the EpiQuik m6A RNA Methylation Quantification Kit (Epigentek; Farmingdale, NY, USA). HepG2.2.15 and HepG2 cells expressing HBx were lysed, and their RNA content was added to binding wells using RNA high-binding solution. Subsequently, appropriate concentrations of capture and detection

antibody solutions were added to the wells. Absorbance at 450 nm was measured using a microplate reader (Bio-Rad; Hercules, CA, USA) to quantify m6A levels based on a colorimetric method, utilizing calculations derived from a standard curve.

RNase MazF digestion assay

Cellular RNAs were digested at the unmethylated ACA site using bacterial single-stranded RNase MazF, while sites with m6A methylation remained uncleaved [9]. Based on MazF's ability to discriminate between 5′-ACA-3′ and 5′-(m6A)CA-3′, we determined the m6A methylation modification site on *MALAT1* at 7265^{A} (GGACA). For qRT-PCR, 1 μL MazF was added to each sample, and MazF– was used as a control. The MazF-correction formula is as follows: % MazF– = $(2^{--\mathrm{CtMazF}\,+}) / (2^{--\mathrm{CtMazF}\,-}) \times 100\%$.

RNA decay assay for *MALAT1* stability

To measure RNA stability, the half-life of *MALAT1* was assessed using an RNA decay assay [10]. Briefly, HepG2.2.15 and HepG2 cells expressing HBx were seeded in six-well plates, treated with 5 μg/ml actinomycin D (ActD) (Sigma-Aldrich; St. Louis, MO, USA), and collected at the indicated time points. The cells were harvested, and RNA was isolated using TRIzol reagent and subjected to qRT-PCR as previously described. The half-life (T1/2) of *MALAT1* was calculated, with *U6* used for normalizing the data.

Cell proliferation assay

The Cell Counting Kit-8 (CCK-8) assay was performed using the kit (Vazyme; Nanjing, Jiangsu, China) according to the manufacturer's instructions. Briefly, HepG2.2.15 and HepG2 cells with high and low expression of *MALAT1* were seeded in 96-well plates at an initial density

of 2 × 10^3cells/well. Then, CCK-8 solution was added to each well at the indicated times and incubated for an additional 2 h at 37 °C. Thereafter, OD450 values were measured using a microplate reader (Bio-Rad).

The 5-Ethynyl-2′-deoxyuridine (EdU) staining assay was performed using a BeyoClick™ EdU-488 Kit (Beyotime) following the manufacturer's protocol. Briefly, HepG2.2.15 and HepG2 cells with high and low expression of *MALAT1* were incubated with EdU (50 nM), fixed with 4% paraformaldehyde, permeabilized with 0.5% Triton X-100, and stained with 1 × Click Additive Solution. Cell nucleus were counterstained using 1 × Hoechst 33,342 (1:1000). The EdU positive cells were photographed and counted under an Olympus FSX100 microscope (Olympus; Tokyo, Japan).

For the colony formation assay, HepG2.2.15 and HepG2 cells with high and low expression of *MALAT1* were seeded into 6-well plates with 500 cells/well for 10–14 days to assess colony formation capacity.

Wound healing, migration, and invasion assay

Wound healing assay were performed as described previously [1]. HepG2.2.15 and HepG2 cells with high and low expression of *MALAT1* were seeded in 6-well plates and scratched with a 200 μL pipette tip when 90% confluency was reached. Original scratch widths were recorded, and the scratch widths of cells after 24h of healing were measured. The percentage of wound healing was calculated using the following formula: (original scratch width − healing scratch width) / (original scratch width) × 100%.

For the migration assay, HepG2.2.15 and HepG2 cells with high and low expression of *MALAT1* (4 × 10^4/well) were resuspended in serum-free medium and added to the upper

chambers, while complete medium supplemented with 20% FBS was added to the lower chamber of the Transwell plate (Corning Life Sciences; Corning, NY, USA). After 36 h of culture, the cells on the upper surface of the membranes were removed with a cotton swab, and the migrated cells on the bottom surface of the upper chambers were fixed with 4% paraformaldehyde for 30 min and stained with Crystal Violet Staining solution (Beyotime) for 30 min. The number of migrated cells was counted in three random visual fields using an inverted microscope (Olympus, Tokyo, Japan).

For the invasion assay, the bottom surface of the Transwell upper chamber was precoated with diluted Matrigel (Corning Life Sciences), and the other steps were similar to the migration assay.

Statistical analysis

Statistical analyses were performed using SPSS 22.0 software (SPSS Inc.; Chicago, IL, USA). GraphPad Prism 8.0 (GraphPad Software; San Diego, CA, USA) was used to plot the data. All quantitative values are expressed as the mean ± standard deviation (SD). Student's *t* test and one-way analysis of variance (ANOVA) were used to assess the significance of differences. $P < 0.05$ was considered statistically significance (*$P < 0.05$, ** $P < 0.01$, and *** $P < 0.001$).

Supplementary References

1. Che L, Du ZB, Wang WH, Wu JS, Han T, Chen YY*, et al.* **Intracellular antibody targeting HBx suppresses invasion and metastasis in hepatitis B virus-related hepatocarcinogenesis via protein phosphatase 2A-B56gamma-mediated dephosphorylation of protein kinase B.** *Cell Prolif.* 2022; **55**: e13304.
2. Che L, Wu JS, Du ZB, He YQ, Yang L, Lin JX*, et al.* **Targeting Mitochondrial COX-2 Enhances Chemosensitivity via Drp1-Dependent Remodeling of Mitochondrial Dynamics in Hepatocellular Carcinoma.** *Cancers (Basel).* 2022; **14**: 821.
3. Yamazaki T, Fujikawa C, Kubota A, Takahashi A, Hirose T. **CRISPRa-mediated NEAT1 lncRNA upregulation induces formation of intact paraspeckles.** *Biochem Biophys Res Commun.* 2018; **504**: 218-24.
4. Maeder ML, Linder SJ, Cascio VM, Fu Y, Ho QH, Joung JK. **CRISPR RNA-guided activation of endogenous human genes.** *Nat Methods.* 2013; **10**: 977-9.
5. Li W, Dong X, He C, Tan G, Li Z, Zhai B*, et al.* **LncRNA SNHG1 contributes to sorafenib resistance by activating the Akt pathway and is positively regulated by miR-21 in hepatocellular carcinoma cells.** *J Exp Clin Cancer Res.* 2019; **38**: 183.
6. Yang Y, Zhao X, Wang Z, Shu W, Li L, Li Y*, et al.* **Nuclear Sensor Interferon-Inducible Protein 16 Inhibits the Function of Hepatitis B Virus Covalently Closed Circular DNA by Integrating Innate Immune Activation and Epigenetic Suppression.** *Hepatology.* 2020; **71**: 1154-69.
7. Li Y, He M, Wang Z, Duan Z, Guo Z, Wang Z*, et al.* **STING signaling activation inhibits HBV replication and attenuates the severity of liver injury and HBV-induced fibrosis.** *Cell Mol Immunol.* 2022; **19**: 92-107.
8. Zhuang Q, Zhou T, He C, Zhang S, Qiu Y, Luo B*, et al.* **Protein phosphatase 2A-B55delta enhances chemotherapy sensitivity of human hepatocellular carcinoma under the regulation of microRNA-133b.** *J Exp Clin Cancer Res.* 2016; **35**: 67.
9. Yan W, Lin X, Ying Y, Li J, Fan Z. **Specific RNA m6A modification sites in bone marrow mesenchymal stem cells from the jawbone marrow of type 2 diabetes patients with dental implant failure.** *Int J Oral Sci.* 2023; **15**: 6.
10. Ma L, Chen T, Zhang X, Miao Y, Tian X, Yu K*, et al.* **The m(6)A reader YTHDC2 inhibits lung adenocarcinoma tumorigenesis by suppressing SLC7A11-dependent antioxidant function.** *Redox Biol.* 2021; **38**: 101801.

Supplementary figures and legends

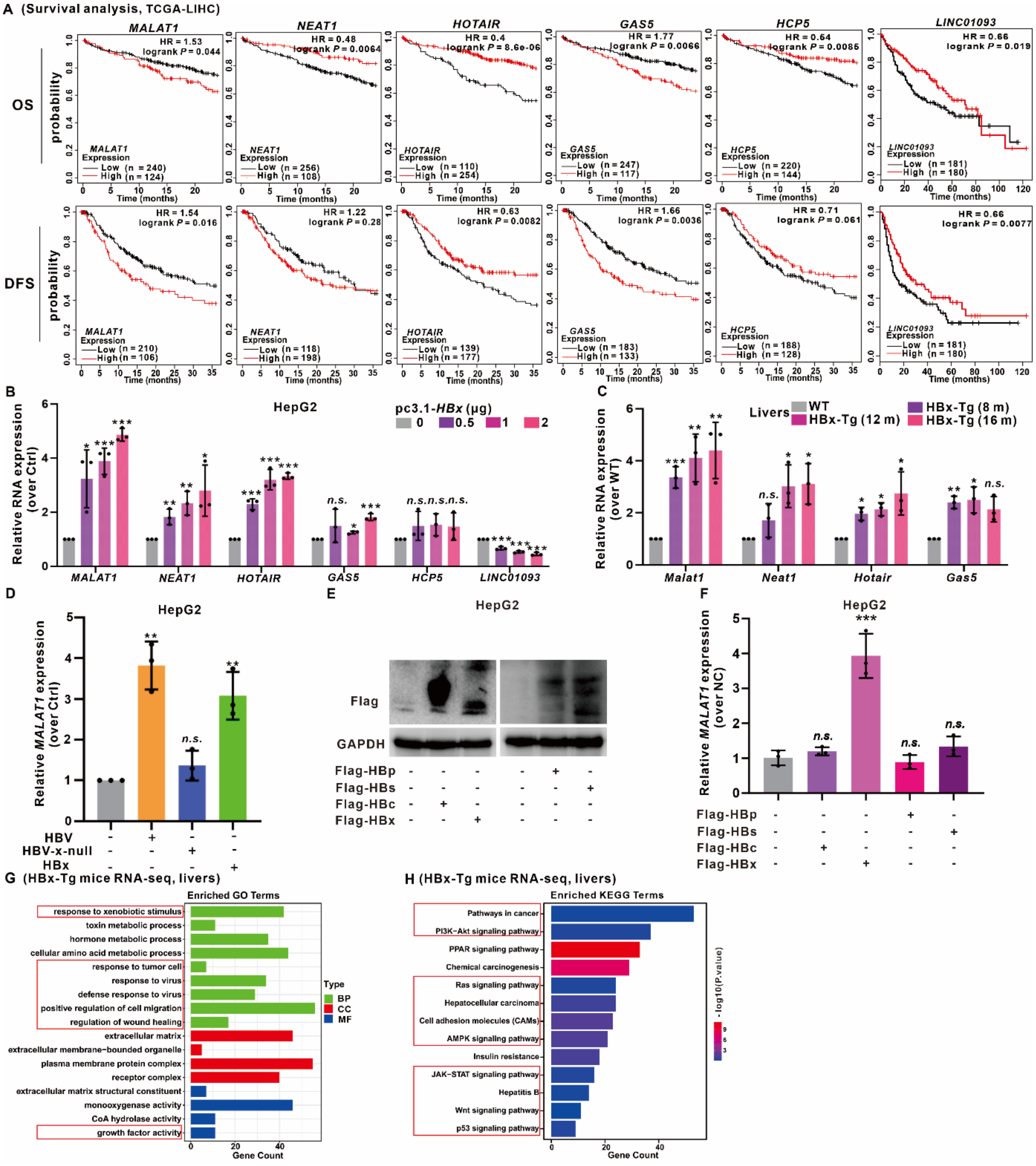

Fig. S1. HBx expression-upregulated *MALAT1* is positively correlated with poor prognosis in HCC patients. A Kaplan-Meier analysis of OS and DFS of patients with *MALAT1*, *NEAT1*, *HOTAIR*, *GAS5*, *HCP5*, and *LINC01093* in the TCGA-LIHC cohort. HR, hazard ratio. **B** Relative expression of the candidate lncRNAs was detected by qRT-PCR in HepG2 cells transfected with different dose of an HBx-expressing

plasmid. **C** Relative expression of the candidate lncRNAs was detected by qRT-PCR in livers from HBx-Tg mice. **D** Relative expression of *MALAT1* was detected by qRT-PCR in HepG2 cells transfected the HBx, HBV, or HBV-x-null plasmids. **E** HepG2 cells were transfected with Flag-tagged HBp, HBs, HBc, and HBx plasmids (1 μg each) for 24 h, with a blank vector used as a negative control (NC). The expression levels of HBp, HBs, HBc, and HBx were detected by WB. **F** The mRNA level of *MALAT1* was detected by qRT-PCR. **G-H** GO and KEGG enrichment analyses were performed on DEGs in livers from HBx-Tg mice (n = 4) and WT mice (n = 4). *$P < 0.05$; **$P < 0.01$; ***$P < 0.001$. *n.s.*, not significant.

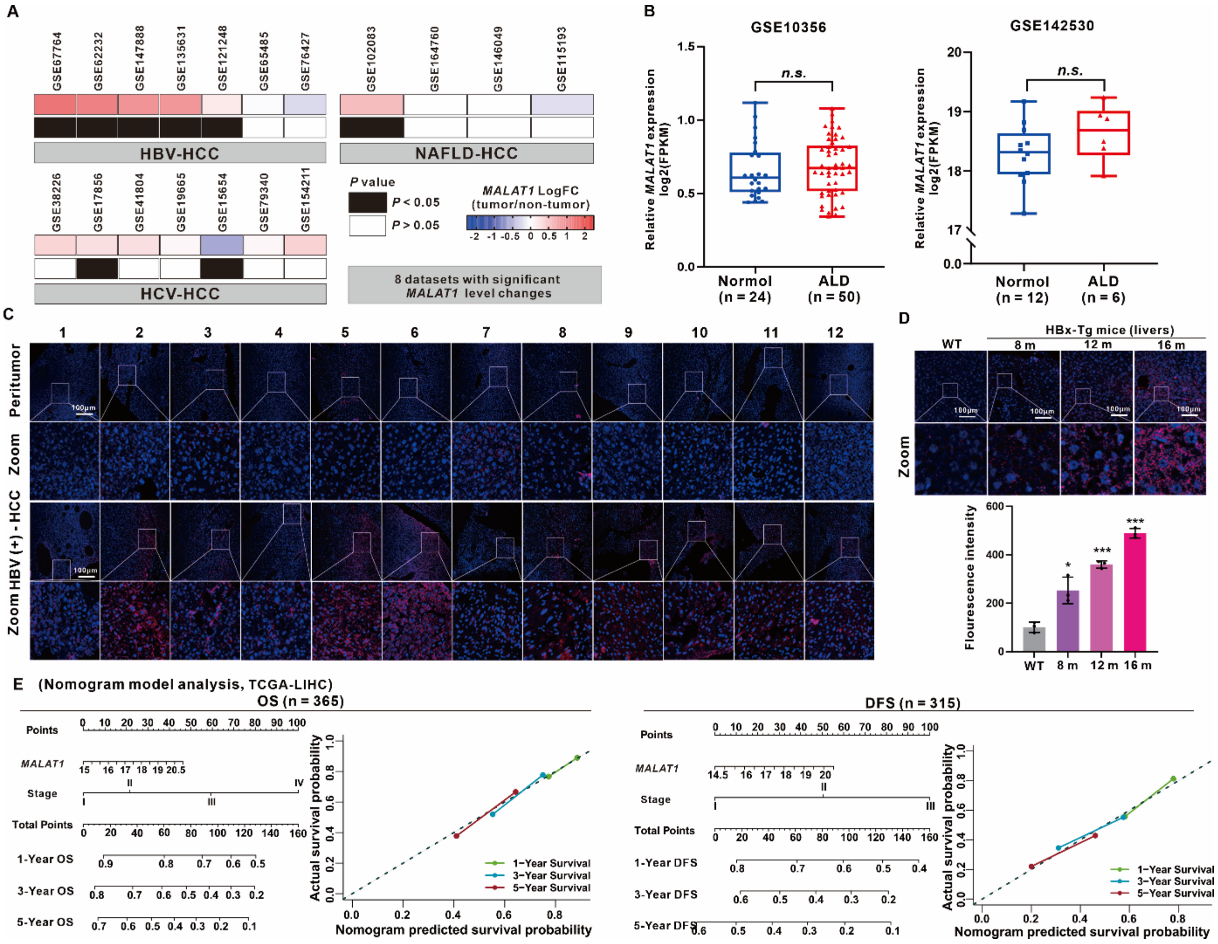

Fig. S2. ***MALAT1*** **is specifically upregulated in HBV-related HCC and correlates with poor prognosis.** **A-B** Comparative analysis of *MALAT1* expression across multiple etiologies of HCC using the GEO database based on large-scale transcriptomic datasets. Publicly available transcriptomic datasets were derived from the GEO database. These additional datasets included *MALAT1* expression data from HBV-related HCC, HCV-related HCC, NAFLD-related HCC **(A)**, and from patients with ALD and normal liver tissues **(B)**. **C-D** The RNA level of *MALAT1* (red) were detected using FISH assays in paired tumor and adjacent peritumor liver tissues from 12 patients with HBV-related HCC patients (C) and in livers from different age HBx-Tg mice (D). Nuclei were visualized by counterstaining with DAPI (blue). Scale bars: 100 μm. **E** A prognostic nomogram was developed to predict the OS and DFS of HCC patients based on the TCGA-LIHC database. The calibration curves of the nomogram demonstrate its accuracy in predicting survival at 1, 3, and 5 years

within the TCGA-LIHC cohort. *$P < 0.05$; **$P < 0.01$; ***$P < 0.001$. *n.s.*, not significant.

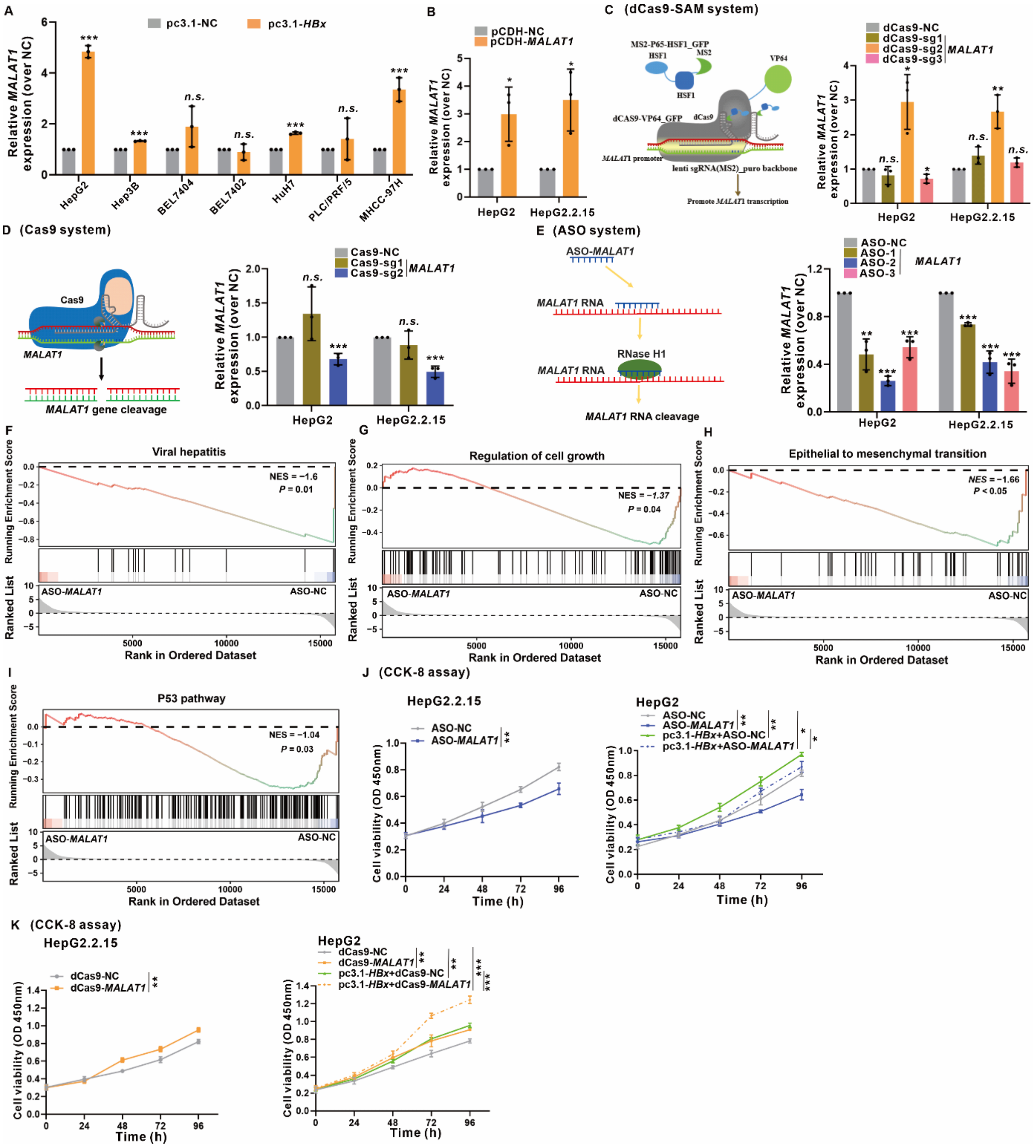

Fig. S3. HBx transcriptionally regulates *MALAT1* expression and *MALAT1* promotes cell proliferation through oncogenic signaling pathways. A Relative expression of *MALAT1* was detected by qRT-PCR in seven HCC cell lines transfected with pc3.1-HBx or the negative control. **B-C** Relative expression of *MALAT1* was detected by qRT-PCR in HepG2 cells and HepG2.2.15 cells transfected with pCDH-*MALAT1*

(B) or dCas9-*MALAT1* **(C)** for stable *MALAT1* overexpression, while the schematic diagram of the dCas9-*MALAT1* system is shown. **D-E** Relative expression of *MALAT1* was detected by qRT-PCR in HepG2 cells and HepG2.2.15 cells transfected with Cas9-*MALAT1* or ASO-*MALAT1* for *MALAT1* knockdown, while the schematic diagrams of the Cas9-*MALAT1* **(D)** and ASO-*MALAT1* **(E)** systems are shown. **F-I** GSEA of DEGs in *MALAT1*-knockdown HepG2.2.15 cells transfected with ASO-*MALAT1* (100 nM, for 24 h) compared to ASO-NC controls. **J** Cell viability was measured using the CCK-8 assays in HepG2.2.15 and HBx-expressing HepG2 cells transfected with dCas9-*MALAT1* (1 μg, for 24 h). **K** Cell viability was measured using the CCK-8 assays in HepG2.2.15 and HBx-expressing HepG2 cells transfected with ASO-*MALAT1* (100 nM, for 24 h). *$P < 0.05$; **$P < 0.01$; ***$P < 0.001$. *n.s.*, not significant.

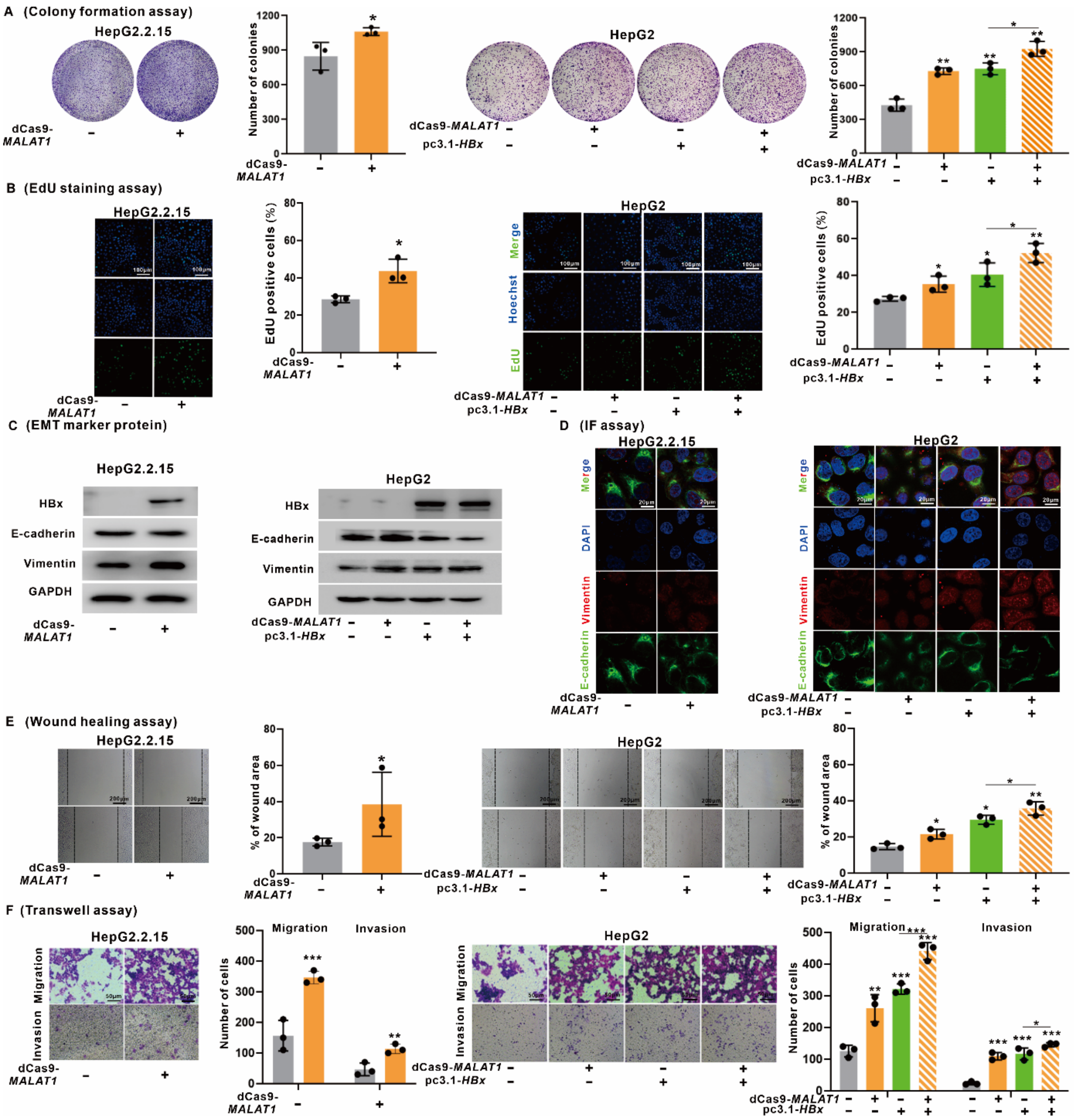

Fig. S4. ***MALAT1*** **promotes the proliferation and metastasis of HBV/HBx-expressing HCC cells** ***in vitro*****.** HepG2.2.15 and HBx-expressing HepG2 cells were transfected with dCas9-*MALAT1* (1 μg, for 24 h) for stable *MALAT1* overexpression, while dCas9-NC was used as a negative control (NC). **A** Colony formation assays were performed to determine the clonogenicity of the cells. The relative number of colonies is shown. **B** Cell proliferation was detected using EdU staining (green). Nuclei were counterstained with Hoechst 33342 (blue). Scale bar: 100 μm. **C-D** The levels of E-cadherin and Vimentin were detected by WB **(C)** and IF

staining **(D)**. Scale bar: 20 μm. **E** Cell migration was measured by wound healing assays. **F** The migration and invasion of the cells were examined via Transwell assays. *$P < 0.05$; **$P < 0.01$; ***$P < 0.001$. *n.s.*, not significant.

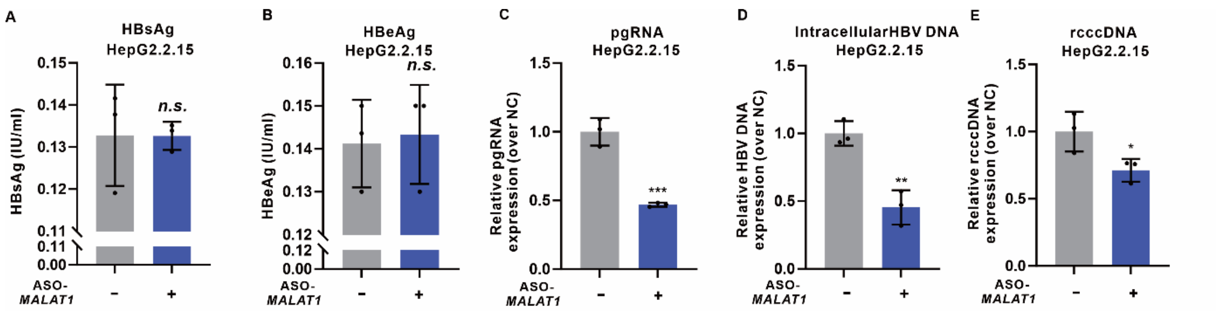

Fig. S5. Knockdown of *MALAT1* suppresses HBV transcription and replication in HepG2.2.15 cells. HepG2.2.15 cells were treated with ASO-*MALAT1* (100 nM) for 24 h, while control oligonucleotides (ASO-NC) were used as a negative control. n = 3. **A-B** Quantification of secreted HBsAg **(A)** and HBeAg **(B)** levels in culture supernatants using ELISA. **C-E** Quantification of HBV pgRNA **(C)**, intracellular HBV DNA **(D)**, and rcccDNA **(E)** was measured by qRT-PCR. *$P < 0.05$; **$P < 0.01$; ***$P < 0.001$. *n.s.*, not significant.

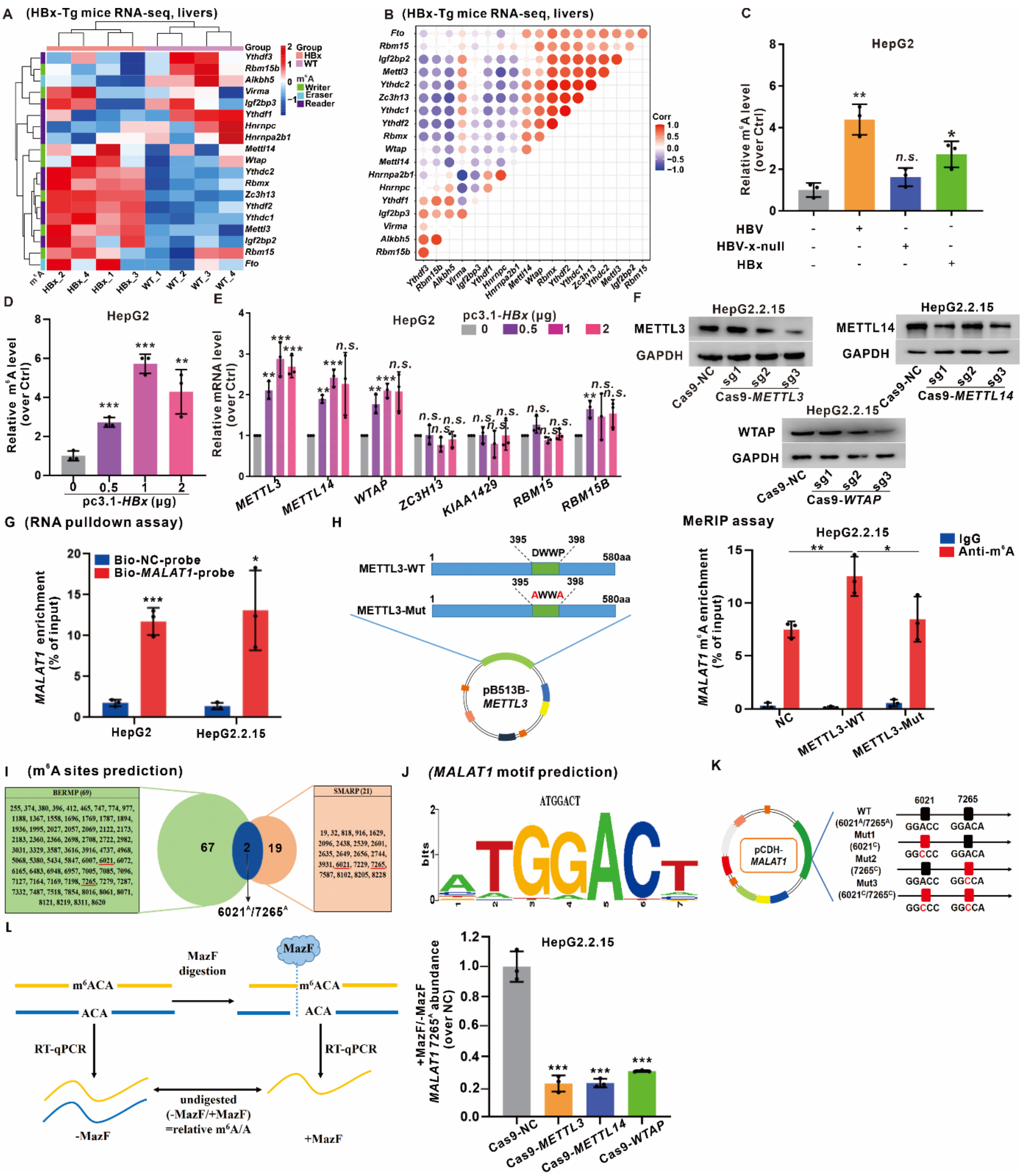

Fig. S6. The m6A level of *MALAT1* is upregulated by the recruitment of the m6A methyltransferase complex. **A** Heatmap of 20 m6A-related genes in the livers of HBx-Tg mice (n = 4) and WT (n = 4). **B** Pearson's correlation analysis was used to determine the relationships between the mRNA levels of 20 m6A-related genes. **C** The overall m6A content in HepG2 cells was analyzed by m6A quantitation analysis after transfection with HBx, HBV, or HBV-x-null plasmids. **D** The overall m6A content in HepG2 cells was

analyzed by m6A quantitation analysis after transfection with different doses of an HBx-expressing plasmid. **E** Relative expression of 7 m6A methyltransferase genes was detected by qRT-PCR in HepG2 cells transfected with different doses of an HBx-expressing plasmid. **F** Protein levels of METTL3, METTL14, and WTAP were detected by WB in HepG2.2.15 cells. **G** RNA pulldown assays were performed to confirm the efficiency of biotin-labeled sense *MALAT1* in HepG2.2.15 and HepG2 cells. **H** METTL3-WT and METTL3-MUT (DWWP to AWWA) plasmids were constructed, and MeRIP analysis of *MALAT1* transcripts was performed in HepG2.2.15 cells transfected with these plasmids. **I** Two databases (BERMP and SMARP) were used to predict the potential m6A sites of *MALAT1*, and a Venn diagram revealed 2 overlapping m6A sites. **J** The m6A-binding motif of *MALAT1* was predicted by RMBase v2.0. **K** Graphic illustration of the construction of WT and m6A site mutant *MALAT1* plasmids. **L** Diagram summarizing the mechanism of MazF RNA endonuclease. MazF cleaves only nonmethylated RNA, sites with m6A methylation remained uncleaved. The abundance of *MALAT1* 7265^{A} was detected by qRT-PCR in HepG2.2.15 cells transfected with indicated plasmids. *$P < 0.05$; **$P < 0.01$; ***$P < 0.001$. *n.s.*, not significant.

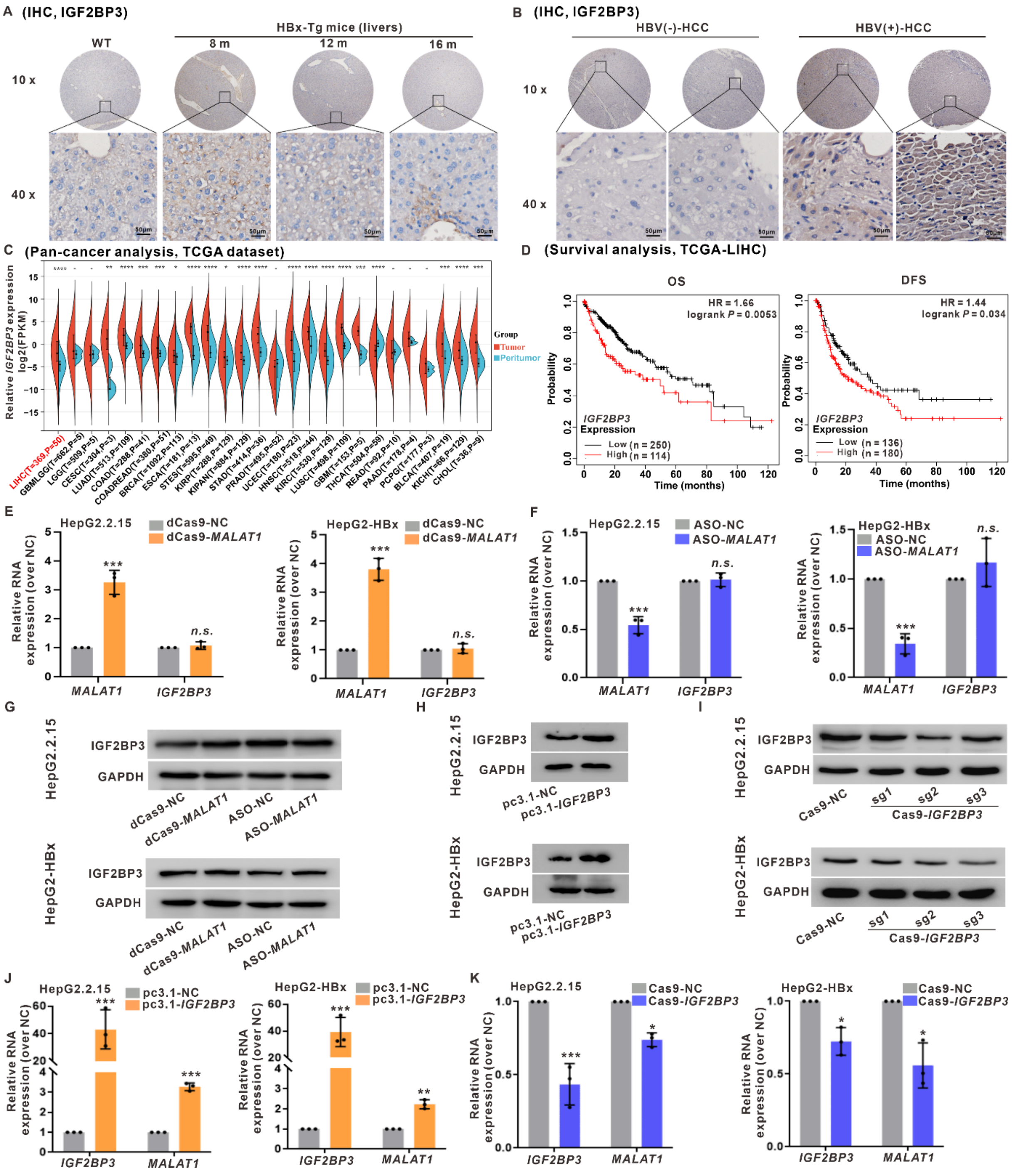

Fig. S7. IGF2BP3 interacts with and stabilizes *MALAT1* in a m6A-dependent manner. A-B Representative images of IGF2BP3 immunostaining in the livers of HBx-Tg mice **(A)** and tissues from patients with HBV-related HCC **(B)**. Scale bars: 50 μm. **C** Pan-cancer analysis of *IGF2BP3* expression in the TCGA-LIHC cohort. **D** Kaplan–Meier analysis of OS and DFS of *IGF2BP3* in the TCGA-LIHC cohort. HR, hazard ratio. **E-F** Relative expression of *MALAT1* and *IGF2BP3* was detected by qRT-PCR in HepG2.2.15

and HBx-expressing HepG2 cells transfected with dCas9-*MALAT1* **(E)** or ASO-*MALAT1* **(F)**. **G** Protein level of IGF2BP3 was detected by WB in HepG2.2.15 and HBx-expressing HepG2 cells transfected with dCas9-*MALAT1* or ASO-*MALAT1*. **H-I** Protein level of IGF2BP3 was detected by WB in HepG2.2.15 and HBx-expressing HepG2 cells transfected with pc3.1-*IGF2BP3* **(H)** or Cas9-*IGF2BP3* **(I)** plasmids. **J-K** Relative expression of *MALAT1* and *IGF2BP3* was detected by qRT-PCR in HepG2.2.15 and HBx-expressing HepG2 cells transfected with pc3.1-*IGF2BP3* **(J)** or Cas9-*IGF2BP3* **(K)** plasmids. *$P < 0.05$; **$P < 0.01$; ***$P < 0.001$. *n.s.*, not significant.

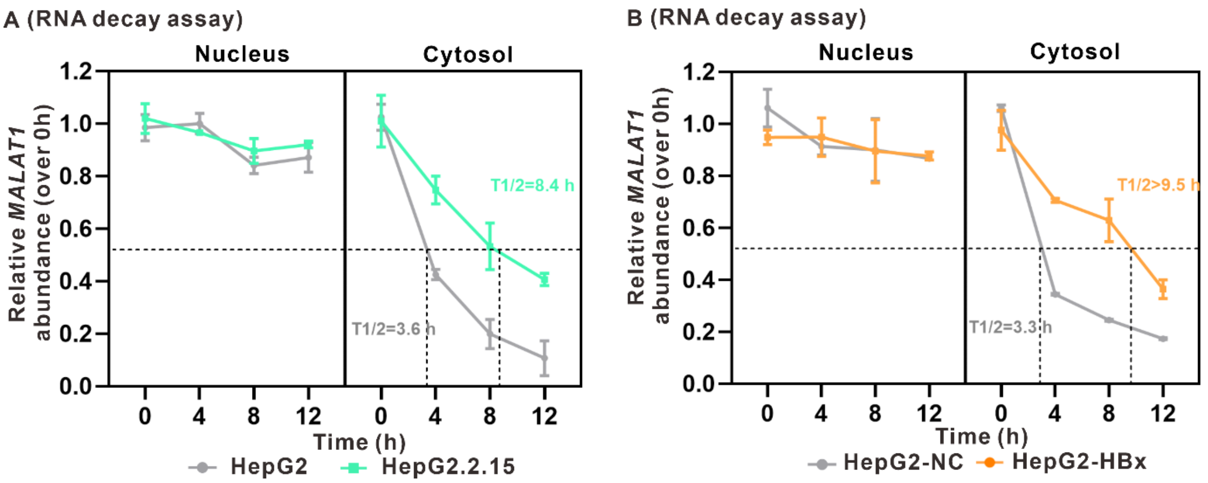

Fig. S8. IGF2BP3 regulates the stability of nuclear and cytoplasmic *MALAT1* RNA in HBV/HBx-expressing HCC cells. HepG2.2.15 cells and HBx-expressing HepG2 cells were used for experiments, whereas HepG2 cells and HepG2-NC cells served as controls. **A-B** RNA decay assays showing the nuclear and cytoplasmic *MALAT1* RNA half-life in HepG2.2.15 cells **(A)** and HBx-expressing HepG2 cells **(B)**.

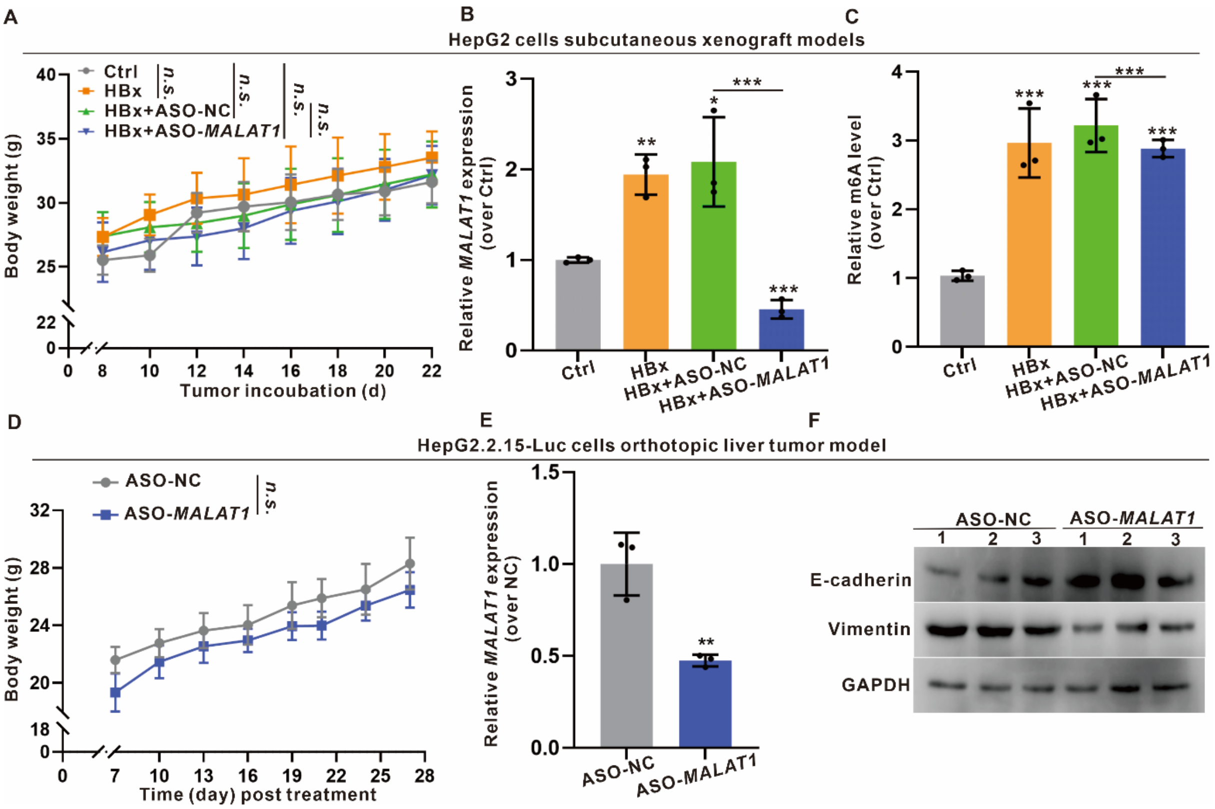

Fig. S9. Targeting *MALAT1 in vivo* with ASO-*MALAT1* treatment effectively suppressed xenograft tumor progression in HBx-related HCC. A-C BALB/c nude mice were used to construct the subcutaneous xenograft models harboring HBx-expressing HepG2 cells. The mice were divided into four groups: Ctrl (HepG2-pc3.1-NC), HBx (HepG2-pc3.1-HBx), HBx+ASO-NC, and HBx+ASO-*MALAT1* (n = 8 per group). **A** The body weights of the nude mice were evaluated. **B** The relative expressions of *MALAT1* in the xenograft tumors was detected by qRT-PCR. **C** The overall m6A content in the xenograft tumors was detected by m6A quantitation analysis. **D-F** An orthotopic liver tumor model was established in nude mice by injecting HepG2.2.15-Luc cells. Mice were then treated with ASO-*MALAT1*, while ASO-NC were used as a negative control. n = 7 per group. **D** The body weights of the nude mice were evaluated. **E** The relative expression of *MALAT1* was detected by qRT-PCR. **F** The levels of E-cadherin and Vimentin were detected by WB. *$P < 0.05$; **$P < 0.01$; ***$P < 0.001$. *n.s.*, not significant.

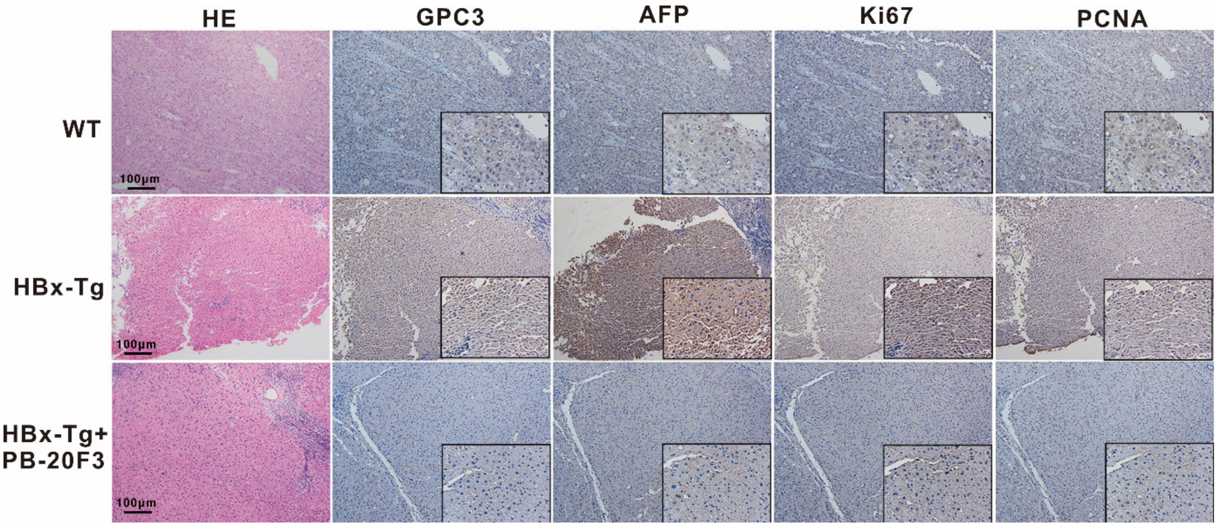

Fig. S10. Anti-HBx intervention suppresses HBx-induced pre-neoplastic features in HBx-Tg mice livers. HBx-Tg mice were used, while wild type (WT) mice served as controls. The anti-HBx monoclonal antibody plasmids (PB-20F3) was delivered via hydrodynamic injection. Representative images showing the HE staining and immunostaining for GPC3, AFP, Ki67, and PCNA in liver tissues from different experimental groups. Scale bars: 100 μm.

Supplementary tables

Table S1. Accession information for datasets retrieved from the GEO database in this study

Accession	GPL	Etiology	Sample size	Source URL
GSE6764	GPL570 Affymetrix Human Genome U133 Plus 2.0 Array	HCV-HCC	45<br>Tumor=35<br>Normal=10	https://www.ncbi.nlm.nih.gov/geo/query/acc.cgi?acc = GSE6764
GSE62232	GPL570 Affymetrix Human Genome U133 Plus 2.0 Array	HBV-HCC	26<br>Tumor=16<br>Normal=10	https://www.ncbi.nlm.nih.gov/geo/query/acc.cgi?acc = GSE62232
GSE98269	GPL21047 Agilent-074348 Human LncRNA v6 4X180K	HCC	6<br>Tumor=3<br>Normal=3	https://www.ncbi.nlm.nih.gov/geo/query/acc.cgi?acc = GSE98269
GSE67764	GPL17077 Agilent-039494 SurePrint G3 Human GE v2 8x60K Microarray 039381 (Probe Name version)	HBV-HCC	6<br>Tumor=3<br>Normal=3	https://www.ncbi.nlm.nih.gov/geo/query/acc.cgi?acc=GSE67764
GSE62232	GPL570 [HG-U133_Plus_2] Affymetrix Human Genome U133 Plus 2.0 Array	HBV-HCC	20<br>Tumor=10<br>Normal=10	https://www.ncbi.nlm.nih.gov/geo/query/acc.cgi?acc=GSE62232
GSE147888	GPL4133 Agilent-014850 Whole Human Genome Microarray 4x44K G4112F (Feature Number version)	HBV-HCC	12<br>Tumor=6<br>Normal=6	https://www.ncbi.nlm.nih.gov/geo/query/acc.cgi?acc=GSE147888
GSE135631	GPL16791 Illumina HiSeq 2500 (Homo sapiens)	HBV-HCC	30<br>Tumor=15<br>Normal=15	https://www.ncbi.nlm.nih.gov/geo/geo2r/?acc=GSE135631
GSE121248	GPL570 [HG-U133_Plus_2] Affymetrix Human Genome U133 Plus 2.0 Array	HBV-HCC	107<br>Tumor=70<br>Normal=37	https://www.ncbi.nlm.nih.gov/geo/geo2r/?acc=GSE121248

GSE65485	GPL11154 Illumina HiSeq 2000 (Homo sapiens)	HBV-HCC	55 Tumor=50 Normal=5	https://www.ncbi.nlm.nih.gov/geo/geo2r/?acc=GSE65485
GSE76427	GPL10558 Illumina HumanHT-12 V4.0 expression beadchip	HBV-HCC	167 Tumor=115 Normal=52	https://www.ncbi.nlm.nih.gov/geo/geo2r/?acc=GSE76427
GSE38226	GPL6947 Illumina HumanHT-12 V3.0 expression beadchip	HCV-HCC	10 Tumor=6 Normal=4	https://www.ncbi.nlm.nih.gov/geo/geo2r/?acc=GSE38226
GSE17856	GPL6480 Agilent-014850 Whole Human Genome Microarray 4x44K G4112F (Probe Name version)	HCV-HCC	87 Tumor=43 Normal=44	https://www.ncbi.nlm.nih.gov/geo/query/acc.cgi?acc=GSE17856
GSE41804	GPL570 [HG-U133_Plus_2] Affymetrix Human Genome U133 Plus 2.0 Array	HCV-HCC	40 Tumor=20 Normal=20	https://www.ncbi.nlm.nih.gov/geo/geo2r/?acc=GSE41804
GSE19665	GPL570 [HG-U133_Plus_2] Affymetrix Human Genome U133 Plus 2.0 Array	HCV-HCC	10 Tumor=5 Normal=5	https://www.ncbi.nlm.nih.gov/geo/geo2r/?acc=GSE19665
GSE15654	GPL8432 Illumina HumanRef-8 WG-DASL v3.0	HCV-HCC	216 Tumor=65 Normal=151	https://www.ncbi.nlm.nih.gov/geo/geo2r/?acc=GSE15654
GSE79340	GPL6244 [HuGene-1_0-st] Affymetrix Human Gene 1.0 ST Array [transcript (gene) version]	HCV-HCC	6 Tumor=3 Normal=3	https://www.ncbi.nlm.nih.gov/geo/geo2r/?acc=GSE79340
GSE154211	GPL11154 Illumina HiSeq 2000 (Homo sapiens)	HCV-HCC	10 Tumor=5 Normal=5	https://www.ncbi.nlm.nih.gov/geo/geo2r/?acc=GSE154211
GSE102083	GPL570 [HG-U133_Plus_2] Affymetrix Human Genome U133 Plus 2.0 Array	NAFLD-HCC	166 Tumor=152 Normal=14	https://www.ncbi.nlm.nih.gov/geo/geo2r/?acc=GSE102083

GSE164760	GPL13667 [HG-U219] Affymetrix Human Genome U219 Array	NAFLD-HCC	59 Tumor=53 Normal=6	https://www.ncbi.nlm.nih.gov/geo/geo2r/?acc=GSE164760
GSE146049	GPL10558 Illumina HumanHT-12 V4.0 expression beadchip	NAFLD-HCC	10 Tumor=5 Normal=5	https://www.ncbi.nlm.nih.gov/geo/geo2r/?acc=GSE146049
GSE115193	GPL16791 Illumina HiSeq 2500 (Homo sapiens)	NAFLD-HCC	9 Tumor=6 Normal=3	https://www.ncbi.nlm.nih.gov/geo/geo2r/?acc=GSE115193
GSE10356	GPL5215 INSERM Homo sapiens 14K array Liverpool3	ALD	74 ALD=50 Normal=24	https://www.ncbi.nlm.nih.gov/geo/geo2r/?acc=GSE10356
GSE142530	GPL11154 Illumina HiSeq 2000 (Homo sapiens)	ALD	18 ALD=6 Normal=12	https://www.ncbi.nlm.nih.gov/geo/query/acc.cgi?acc=GSE142530

Table S2. Sequences of primers for various plasmid constructions in this study

**Plasmids**	**Primers**	**Sequences** (5'-3')
Cas9-*MALAT1*-sg1	FP	CACCGAAATGGCGCTGCGCTTAAGA
	RP	AAACTCTTAAGCGCAGCGCCATTTC
Cas9-*MALAT1*-sg2	FP	CACCGGATAGTACACTTCACTCAG
	RP	AAACCTGAGTGAAGTGTACTATCC
Cas9-*MALAT1*-sg3	FP	CACCGCAGCAGACACACGTATGCGA
	RP	AAACTCGCATACGTGTGTCTGCTGC
Cas9-*IGF2BP3*-sg1	FP	CACCGTATCCCGCCTCATTTACAGT
	RP	AAACACTGTAAATGAGGCGGGATAC
Cas9-*IGF2BP3*-sg2	FP	CACCGAATCACGATATCTCCATTGC
	RP	AAACGCAATGGAGATATCGTGATTC
Cas9-*IGF2BP3*-sg3	FP	CACCGATACTTTCTAGGTCCGAGG
	RP	AAACCCTCGGACCTAGAAAGTATC
Cas9-*METTL3*-sg1	FP	CACCGATCATTCGGACAGGCCGTAC
	RP	AAACGTACGGCCTGTCCGAATGATC
Cas9-*METTL3*-sg2	FP	CACCGCTGCAACGCATCATTCGGAC
	RP	AAACGTCCGAATGATGCGTTGCAGC
Cas9-*METTL3*-sg3	FP	CACCGCTCAACATACCCGTACTAC
	RP	AAACGTAGTACGGGTATGTTGAGC
Cas9-*METTL14*-sg1	FP	CACCGAGAGATAGTACAATTCGACC
	RP	AAACGGTCGAATTGTACTATCTCTC

Cas9-*METTL14*-sg2	FP	CACCGTAGTTGCTATTTGTAAGCGT
	RP	AAACACGCTTACAAATAGCAACTAC
Cas9-*METTL14*-sg3	FP	CACCGATAGTACAATTCGACCAGGT
	RP	AAACACCTGGTCGAATTGTACTATC
Cas9-*WTAP*-sg1	FP	CACCGCTTGGGAAGAGGTTCTTCGT
	RP	AAACACGAAGAACCTCTTCCCAAGC
Cas9-*WTAP*-sg2	FP	CACCGCGAAGAACCTCTTCCCAAGA
	RP	AAACTCTTGGGAAGAGGTTCTTCGC
Cas9-*WTAP*-sg3	FP	CACCGTGTAATGCGACTAGCAACCA
	RP	AAACTGGTTGCTAGTCGCATTACAC
dCas9-*MALAT1*-sg1	FP	CACCGGATAGTACACTTCACTCAG
	RP	AAACCTGAGTGAAGTGTACTATCC
dCas9-*MALAT1*-sg2	FP	CACCGCAGTGCCTTGATGCCAACTA
	RP	AAACTAGTTGGCATCAAGGCACTGC
dCas9-*MALAT1*-sg3	FP	CACCGTAAATAACCTCTTAGACAGG
	RP	AAACCCTGTCTAAGAGGTTATTTAC
pc3.1-*IGF2BP3*	FP	CGGGGTACCATGAACAAACTGTATATCGG
pc3.1-*IGF2BP3*(1-78aa)	RP	CGGAATTCTTACTTATCGTCGTCATCCTTGTAATCTTGCCTTTTTGGGACCG
pc3.1-*IGF2BP3* (1-157aa)	RP	CGGAATTCTTACTTATCGTCGTCATCCTTGTAATCTTCATCAGGGATATAGG
pc3.1-*IGF2BP3* (1-263aa)	RP	CGGAATTCTTACTTATCGTCGTCATCCTTGTAATCAATCTCCAGAATAGACT
pc3.1-*IGF2BP3* (1-345aa)	RP	CGGAATTCTTACTTATCGTCGTCATCCTTGTAATCCTTCATGATCTCCTCCT
pc3.1-*IGF2BP3* (1-472aa)	RP	CGGAATTCTTACTTATCGTCGTCATCCTTGTAATCTCCATAAATTCTTCCCT
pc3.1-*IGF2BP3* (1-577aa)	RP	CGGAATTCTTACTTATCGTCGTCATCCTTGTAATCCTTCCGTCTTGACTGAG
PB513B-*METTL3*	FP	CTAGCTAGCGCCACCATGTCGGACACGTGGAGCTCTATC
	RP	ATAAGCATGCGGCCGCCTATAAATTCTTAGGTTTAGAGATGATAC
PB513B-*METTL3* (D395A-P398A)	FP	TGTGATGGCTGCCCCACCCGCGGATATTCACATGGAACTGCCCTATGGG
	RP	ATGTGAATATCCGCGGGTGGGGCAGCCATCACAACTGCAAACTTGCCC
pCDH-*MALAT1*-Mut1	FP	CCCAGATCAGGATTTGAGCGGAAGAAC
	RP	CCATTAAAGAGTGTTCGCAGACAAAGTT
pCDH-*MALAT1*- Mut2	FP	CCAAAATGAGGAAAACAGGTGAACAAG
	RP	CCACTGGTGAATTCAACTGGAAGCTCC
ASO-*MALAT1*-1		ACUUAACUUCAACAUUUGGCC
ASO-*MALAT1*-2		AAUUAAUGCACUGGUACACCC

ASO-*MALAT1*-3	UUUAUUCCUUAUUUAGAGGGC
ASO-NC	UUCUCCGAACGUGUCACGUTT

Table S3. Sequences of primers for qRT-PCR analysis in this study

**Genes**	**Primers**	**Sequences** (5'-3')
MALAT1	FP	ACGCAGCGACGAGTTG
	RP	TGCCTGGTTAGGTATGAGC
NEAT1	FP	GGTGGCAGTGCTCCTT
	RP	CATTACCAACAATACCGACT
HOTAIR	FP	GGTAGAAAAAGCAACCACGAAGC
	RP	ACATAAACCTCTGTCTGTGAGTGCC
GAS5	FP	TATGGTGCTGGGTGCGGAT
	RP	CCAATGGCTTGAGTTAGGCTT
HCP5	FP	GCTGGACGATTCTCCTCACACT
	RP	CTCCTCTCCAGGCACAGGTAAT
LINC01093	FP	GGTCACTATGTTACACCTGC
	RP	TGCTGTCCTCAGGGCAAGCT
Malat1	FP	TTGGCTTGTCAACTGCG
	RP	AGGAATGTTACCGCACC
Neat1	FP	ATGTCTTGTTCTGGGAGC
	RP	CCTTACGCAATCTTCTCG
Hotair	FP	CCTTATAAGCTCATCGGAGCA
	RP	CATTTCTGGGTGGTTCCTTT
Gas5	FP	GGATAACAGAGCGAGCGCAAT
	RP	CCAGCCAAATGAACAAGCATG
METTL3	FP	CTATCTCCTGGCACTCGCAAGA
	RP	GCTTGAACCGTGCAACCACATC
METTL14	FP	CTGAAAGTGCCGACAGCATTGG
	RP	CTCTCCTTCATCCAGATACTTACG
WTAP	FP	GCAACAACAGCAGGAGTCTGCA
	RP	CTGCTGGACTTGCTTGAGGTAC
ZC3H13	FP	CGGACAGTGATGCCTACAACAG
	RP	TCTGTGAGGTGCGAGGGACTAA
KIAA1429	FP	TGACCTTGCCTCACCAACTGCA
	RP	AGCAACCTGGTGGTTTGGCTAG
RBM15	FP	CTTCCCACCTTGTGAGTTCTCC
	RP	CTTCTTGTTCTCATACCTAACTCC
RBM15B	FP	TGGTAACCTGGACCACAGCGTA
	RP	GGTTCTGGAACTTGAGGAAGGC
IGF2BP1	FP	CAGGAGATGGTGCAGGTGTTTATCC
	RP	GTTTGCCATAGATTCTTCCCTGAGC
IGF2BP2	FP	AGTGGAATTGCATGGGAAAATCA

	RP	CAACGGCGGTTTCTGTGTC
IGF2BP3	FP	AGTTGTTGTCCCTCGTGACC
	RP	GTCCACTTTGCAGAGCCTTC
ZFP36	FP	GCTATGTCGGACCTTCTCAGAG
	RP	CCTGGAGGTAGAACTTGTGACAG
LIN28B	FP	CCTGTTTAGGAAGTGAAAGAAGAC
	RP	CACTTCTTTGGCTGAGGAGGTAG
MSI1	FP	GCTCAGCCAAAGGAGGTGATGT
	RP	GCGTAGGTTGTGGCTTGGAAAC
U6	FP	CTCGCTTCGGCAGCACA
	RP	AACGCTTCACGAATTTGCGT
GAPDH	FP	GTCTCCTCTGACTTCAACAGCG
	RP	ACCACCCTGTTGCTGTAGCCAA
Gapdh	FP	CATCACTGCCACCCAGAAGACTG
	RP	ATGCCAGTGAGCTTCCCGTTCAG
pgRNA	FP	TGTTCAAGCCTCCAAGCT
	RP	GGAAAGAAGTCAGAAGGCAA
HBV DNA	FP	CCCGTTTGTCCTCTAATTCC
	RP	GTCCGAAGGTTTGGTACAGC
cccDNA	FP	CTCCCCGTCTGTGCCTTCT
	RP	GCCCCAAAGCCACCCAAG

Table S4. Information on the primary antibodies used in the present study

**Antibody**	**Source**	**Catalog No.**	**Application (Dilution)**
HBx (anti-mouse)	Santa Cruz	sc-57760	WB (1:1000); IHC (1:200)
METTL3 (anti-rabbit)	Abcam	ab195352	WB (1:1000); IF (1:400); IP (1:50); IHC (1:200)
METTL14 (anti-rabbit)	Proteintech	26158-1-AP	WB (1:2000); IHC (1:200)
WTAP (anti-rabbit)	Abcam	ab195380	WB (1:1000) ; IHC (1:200)
IGF2BP3 (anti-rabbit)	Abclonal	A23295	WB (1:1000) ; IHC (1:200) RIP (5 μg)
E-cadherin (anti-rabbit)	CST	24E10	WB (1:1000); IF (1:400) IHC (1:400)
Vimentin (anti-rabbit)	R28	3932	WB (1:1000) ; IHC (1:200)
Ki67 (anti-mouse)	D3B5	9129	IHC (1:400)
PCNA (anti-mouse)	Abcam	ab29	IHC (1:200)

GPC3 (anti-rabbit)	Abcam	ab207080	IHC (1:200)
AFP (anti-mouse)	CST	39971	IHC (1:100)
GAPDH (anti-mouse)	CST	14C10	WB (1:1000); IF (1:200)
m6A (anti-rabbit)	Abcam	ab151230	MeRIP (5 μg)
Histone H3 (anti-mouse)	Proteintech	68345-1-Ig	WB (1:5000)
Flag tag (anti-mouse)	Proteintech	66008-4-Ig	WB (1:5000); IP (4.0 μg)

Table S5. Sequences of oligonucleotides for FISH assay and RNA pulldown in this study

**Target gene**	**sense** (5'-3')
MALAT1-FISH	CCGGGGGTTTTGTGAGGTGTTTGATG
MALAT1-pulldown 1	CAGGCTGGTTATGACTCAGAAGATG
MALAT1-pulldown 2	CTCATCAGTAGTAAGAATCTCAGGG
MALAT1-Pulldown 3	CTTCAACAATCACTACTCCAAGCAT
MALAT1-pulldown NC	CCCTGAGATTCTTACTACTGATGAG
